# Improving Uptake of a National Web-Based Psychoeducational Workshop for Informal Caregivers of Veterans: Mixed Methods Implementation Evaluation

**DOI:** 10.2196/16495

**Published:** 2021-01-07

**Authors:** Veronica Yank, Randall C Gale, Andrea Nevedal, Leonore Okwara, Christopher J Koenig, Ranak B Trivedi, Nancy J Dupke, Margaret Kabat, Steven M Asch

**Affiliations:** 1 Division of General Internal Medicine Department of Medicine University of California San Francisco San Francisco, CA United States; 2 Center for Innovation to Implementation VA Palo Alto Health Care System Palo Alto, CA United States; 3 Department of Behavioral and Community Health University of Maryland Baltimore, MD United States; 4 Department of Communication Studies San Francisco State University San Francisco, CA United States; 5 Department of Psychiatry and Behavioral Sciences Stanford University Palo Alto, CA United States; 6 VA Caregivers Support Program Department of Veterans Affairs Washington, DC United States; 7 Division of Primary Care and Population Health Stanford University Palo Alto, CA United States

**Keywords:** web-based workshop, implementation science, behavioral intervention technology, informal caregiver, family caregiver, veteran, psychoeducation, self-management, outreach, marketing

## Abstract

**Background:**

Although web-based psychoeducational programs may be an efficient, accessible, and scalable option for improving participant well-being, they seldom are sustained beyond trial publication. Implementation evaluations may help optimize program uptake, but few are performed. When the US Department of Veterans Affairs (VA) launched the web-based psychoeducational workshop Building Better Caregivers (BBC) for informal caregivers of veterans nationwide in 2013, the workshop did not enroll as many caregivers as anticipated.

**Objective:**

This study aims to identify the strengths and weaknesses of initial implementation, strategies likely to improve workshop uptake, whether the VA adopted these strategies, and whether workshop enrollment changed.

**Methods:**

We used mixed methods and the Promoting Action on Research Implementation in Health Services (PARIHS) implementation evaluation framework. In stage 1, we conducted semistructured interviews with caregivers, local staff, and regional and national VA leaders and surveys with caregivers and staff. We collected and analyzed survey and interview data concurrently and integrated the results to identify implementation strengths and weaknesses, and strategies likely to improve workshop uptake. In stage 2, we reinterviewed national leaders to determine whether the VA adopted recommended strategies and used national data to determine whether workshop enrollment changed over time.

**Results:**

A total of 54 caregivers (n=32, 59%), staff (n=13, 24%), and regional (n=5, 9%) and national (n=4, 7%) leaders were interviewed. We received survey responses from 72% (23/32) of caregivers and 77% (10/13) of local staff. In stage 1, survey and interview results were consistent across multiple PARIHS constructs. Although participants from low-enrollment centers reported fewer implementation strengths and more weaknesses, qualitative themes were consistent across high- and low-enrollment centers, and across caregiver, staff, and leadership respondent groups. Identified strengths included belief in a positive workshop impact and the use of some successful outreach approaches. Implementation weaknesses included missed opportunities to improve outreach and to better support local staff. From these, we identified and recommended new and enhanced implementation strategies—increased investment in outreach and marketing capabilities; tailoring outreach strategies to multiple stakeholder groups; use of campaigns that are personal, repeated, and detailed, and have diverse delivery options; recurrent training and mentoring for new staff; and comprehensive data management and reporting capabilities. In stage 2, we determined that the VA had adopted several of these strategies in 2016. In the 3 years before and after adoption, cumulative BBC enrollment increased from 2139 (2013-2015) to 4030 (2016-2018) caregivers.

**Conclusions:**

This study expands the limited implementation science literature on best practices to use when implementing web-based psychoeducational programs. We found that robust outreach and marketing strategies and support for local staff were critical to the implementation success of the BBC workshop. Other health systems may want to deploy these strategies when implementing their web-based programs.

## Introduction

### Background

Evidence-based online psychoeducational programs may offer an efficient, accessible, and scalable option for improving participants’ health and well-being [1-9] but only if they succeed in reaching the participants for whom they are designed [10,11]. Unfortunately, few web-based interventions are widely implemented following study completion [3,12]. Programs for informal caregivers offer a case in point [13]. Informal caregivers are family members, friends, or acquaintances (hereafter referred to as *caregivers*) who provide essential support to persons living with major health conditions or disabilities in the community [14,15]. Although web-based psychoeducational programs for caregivers have proliferated, the best strategies for optimizing their implementation remain unknown [7,16-19].

Among caregivers, those providing support to US veterans have some of the highest documented rates of caregiving burden, stress, depression, and neglect of self-care [20,21]. They have expressed a desire for help from the US Department of Veterans Affairs (VA) Health Care System, which is the largest integrated health care system in the United States [20]. Galvanized by a 2010 Act of the US Congress [22], the VA Caregiver Support Program (CSP) commissioned the development of a web-based psychoeducational and self-management workshop, *Building Better Caregivers* (BBC), for these caregivers. In earlier studies, BBC participants experienced reduced stress and depression and increased self-efficacy [23,24]. On the basis of this evidence, the VA adopted the BBC workshop nationally in 2013 at all VA medical centers in the United States and its territories [22]. However, BBC program uptake was lower than expected at many medical centers.

### Objectives

We seek to determine why the national web-based BBC workshop did not reach caregivers at some centers and whether uptake could be improved by performing a multistage mixed methods implementation evaluation involving national and regional VA leadership, staff and informal caregivers at local centers, and national VA CSP operations data sets. Specifically, our objectives were organized into 2 stages:

*Stage 1: initial evaluation.*

- 1a. Identify initial implementation strengths and weaknesses.
- 1b. Identify and recommend implementation strategies likely to improve uptake.

*Stage 2: follow-up evaluation.*

- 2a. Determine whether new or enhanced implementation strategies were subsequently adopted.
- 2b. Determine whether workshop enrollment changed.

## Methods

### Study Design

We employed a convergent mixed methods design for both stages of research because the implementation of a web-based caregiver workshop nationally among VA medical centers was a complex health services intervention that required multilevel processes, which we felt would be best assessed with a mixed methods approach [25]. We used the revised Promoting Action on Research Implementation in Health Services (PARIHS) framework as our implementation evaluation framework [26,27]. The PARIHS constructs include (1) evidence (eg, research evidence supporting innovation effectiveness, participant experiences); (2) context (eg, resources necessary to support staff activities, data management and evaluation capabilities); (3) facilitation (eg, skills and attributes of local staff, staff training, and mentoring and assistance from experts); and (4) successful—or unsuccessful—implementation (eg, achievement of desired outcomes of innovation, extent of uptake). These PARIHS constructs were represented throughout our mixed methods process, including in the structure of our sampling frame, survey questions, interview questions, and the analysis and interpretation of both quantitative and qualitative data. The Stanford University Institutional Review Board approved the evaluation (protocol no. 29965), and participants provided verbal consent. Caregivers received US $40 as compensation for study participation. The staff and leaders did not receive compensation.

### Stage 1: Identification of Initial Implementation Strengths and Weaknesses and Strategies Likely to Improve Workshop Uptake

In stage 1, we collected and assessed survey and interview data concurrently to enable planned triangulation between the data sets and results (October 2014 to January 2015). The qualitative (interview) approach was the higher priority approach because it enabled the collection of detailed, nuanced feedback on implementation strengths and weaknesses and suggestions for changes not achievable in a survey. However, the quantitative (survey) approach was integral to the implementation evaluation because it enabled anonymized structured feedback on prespecified, discrete implementation elements derived from the PARIHS constructs and subconstructs that might or might not be mentioned during interviews and that we could compare and integrate with emergent themes from the qualitative data. Surveys also generated contextualizing data on respondent characteristics (eg, age, caregiving relationship, care partner health conditions). The quantitative and qualitative analyses were performed by the study team, and the study team, the advisory board, and a national VA CSP leader integrated findings and reached consensus on strategies likely to improve workshop uptake during a half-day virtual retreat (May 2015). The study team communicated findings and recommendations to VA CSP national leaders in a final report and webinar meetings (June to July 2015).

### Characteristics of the Workshop

The BBC workshop is an evidence-based, 6-week, interactive psychoeducational and self-management program designed to reduce caregiver stress and depression and increase caregiver skills and self-efficacy [23,24]. Each workshop includes 20-25 participants and is guided by 2 trained cofacilitators. During workshops, caregivers read weekly lessons, perform self-management- and caregiving-related activities to solidify new skills and interact with and receive social support from peers and facilitators. Examples of self-management and caregiving skills include techniques for stress reduction, creating weekly action plans that are realistic and achievable, problem solving when challenges occur, managing difficult care partner behaviors, communicating with family, friends, and health professionals, and planning for the future, among others. The workshop is delivered on a secure online platform developed specifically for the workshop that, at the time, supported use on a computer or tablet device and has since expanded to include smartphone delivery. The workshop is only available in English.

Workshop facilitators are caregivers of persons with chronic conditions. They receive a 5-day training that teaches them facilitation skills and about the workshop (its theoretical basis, structure, and content). Facilitators must demonstrate mastery of the material and adhere to a comprehensive workshop facilitator manual. Master facilitators monitor each workshop for fidelity. Facilitators can lead workshops remotely from anywhere in the United States and are paid to do so.

### Implementation Evaluation Context and Advisory Board

The VA adopted the BBC workshop nationally in 2013, making it available at no cost to caregivers registered at any VA medical center in the United States or its territories [22]. The VA CSP leadership and our study team at the VA Center for Innovation to Implementation identified that BBC uptake was lower than expected. Using CSP operations data, we determined that 5.74% (1265/22,022) of eligible caregivers enrolled in the workshop in the first 2 years of the rollout, but that center-specific enrollment rates varied widely—from 0% to 37%—with 10.6% (15/142) of all the centers enrolling no participants. Thus, the VA CSP and our study team partnered to perform the current implementation evaluation. As the study team, we convened an advisory board composed of representatives of key stakeholder groups and experts to advise us at all stages of the evaluation. The advisory board included a veteran caregiver, veteran care partner, expert on informal caregiver research, expert on self-management research, clinical psychologist with expertise in eHealth interventions for veterans and their families, and director of web services for the VA.

### Eligibility Criteria and Sample Selection

All 4 national leaders involved in the workshop rollout were invited to participate in the implementation evaluation and all agreed to do so. National leaders were defined as those overseeing or directly managing the BBC implementation at the national level—for example, the VA CSP director, national VA BBC program manager, and BBC project director at the national organization contracted to administer the workshop on behalf of the VA. To identify regional leaders, local staff, and caregivers for participation, we used stratified purposive sampling (Table 1). BBC enrollment rates at all VA medical centers (standardized by catchment size) were calculated to identify high- and low-enrollment centers and the regional networks to which they belonged. At the time, there were 142 medical centers belonging to 21 regional networks. Low-enrollment centers were defined as those in the 10th percentile of referrals during the first 2 years of workshop rollout, and high-enrollment centers were those in the 90th percentile. We identified 5 regional networks that encompassed both low- and high-enrollment centers and invited their CSP regional leaders to participate and all agreed. We then selected 2 regions for local evaluation based on geographical diversity and variation in medical center catchment size: the Northeast region with 2 paired medium-sized centers (1 high-enrollment center and 1 low-enrollment center) and the Gulf Coast with 2 paired large centers (1 high-enrollment center and 1 low-enrollment center). At selected centers, we recruited local staff most involved in the workshop rollout—all CSP social workers who worked as caregiver support coordinators at each center (hereafter referred to as *coordinators*) and other staff whom they identified as involved in the BBC implementation. Among the 14 local staff members approached, 13 (93%) agreed to participate. The staff member who declined had been a substitute coordinator for a short period but had returned to unrelated prior duties by the time we contacted her.

**Table 1:** Participant sampling frame and subsequent enrollment.

Participant characteristics		Sampling frame			Enrollment
		Nation (United States)	Region	Locality	
**Targeted characteristics**					
	Role	National leaders of BBC^a^ rollout (all)	Regional leaders (subset)	Local staff and caregivers (subset)	N/A^b^
	Setting	2 national offices	5 regional networks	4 medical centers	N/A
	N/A	Both in Washington, DC	N/A	N/A	4 national leaders
	N/A	N/A	West	N/A	1 regional leader
	N/A	N/A	Midwest	N/A	1 regional leader
	N/A	N/A	South	N/A	1 regional leader
	N/A	N/A	Gulf Coast	2 large centers^c^	1 regional leader; 9 staff; 16 caregivers
	N/A	N/A	Northeast	2 medium centers^c^	1 regional leader; 4 staff; 16 caregivers
Final sample		N/A	N/A	N/A	54 total participants

^a^BBC: Building Better Caregivers.

^b^N/A: not applicable.

^c^Within the regions selected for additional evaluation, we identified and included one high-enrollment medical center and one low-enrollment medical center matched by catchment size—large size in the Gulf Coast and medium size in the Northeast.

We also recruited 8 veteran caregivers per center (32 in total). Caregiver eligibility criteria were being registered with the VA as a veteran caregiver and being 18 years of age or older. Exclusion criteria included having limited English proficiency and inability to speak by phone for a telephone interview (eg, due to hearing loss). We used VA operations data to identify all registered caregivers at each center and cross-referenced these with operations data on workshop referrals and enrollments. Sampling was divided into target groups of (1) caregivers enrolled in the workshop, (2) caregivers aware of the workshop but not enrolled, and (3) caregivers unaware of the workshop. We randomly generated the order of attempted contact with caregivers and performed a maximum of 2 telephone calls per caregiver. We attempted to contact 163 caregivers and, of these, 10 (6.1%) had a nonworking phone number and 101 (62.0%) did not respond to repeated attempts. We spoke with 54 (33.1%) caregivers. Among these, <1% (1/163) was ineligible because of limited English proficiency, 1.8% (3/163) called us back and desired to enroll but could not because we had reached our enrollment targets, 11.0% (18/163) actively refused, and 19.6% (32/163) enrolled. Reasons for refusal included no time, having no one to take care of veteran or other household responsibilities during the interview, veteran having passed away, being 39 weeks pregnant, and wanting to answer questions by mail only. All potential participants (caregivers, staff, and leaders) received information on the study objectives and researcher identities before their agreement to participate.

### Survey Approach and Data Reporting

The quantitative evaluation consisted of surveys of caregivers and local staff. Caregivers completed paper-based surveys within a week of interview completion (Multimedia Appendix 1). Questions were asked about their demographic and caregiving characteristics and veteran care partners’ demographic and health characteristics. Caregivers who participated in the workshop also indicated whether they would recommend it to others and whether it had improved their quality of life using Likert scale response options 1 to 5 that were anchored by descriptors—strongly disagree, disagree, neither disagree or agree, agree, and strongly agree—and the option of answering do not know/not applicable. The staff survey questions were adapted from the Organizational Readiness to Change Assessment (ORCA), a validated implementation sciences survey structured according to PARIHS constructs (Multimedia Appendix 2) [28]. An additional survey question asked respondents to indicate whether, “At our facility, implementation of the Building Better Caregivers program has been a success,” using Likert scale response options 1 to 5 anchored by descriptors—strongly disagree, disagree, neither disagree or agree, agree, and strongly agree—and the option of answering “do not know/not applicable.” The ORCA is designed for local staff within organizations implementing evidence-based programs. Therefore, it was completed by local staff only (ie, *not* by regional or national leaders). As survey respondent numbers are small, we report results with descriptive statistics rather than comparative statistics—specifically, the number (%) of respondents who gave the response of interest.

### Interview Approach and Data Analysis

The qualitative team consisted of 4 coinvestigators, (RG, CK, AN, and VY) with training and expertise in interview-based data collection and analysis as well as content expertise in the following areas: public health and palliative care (RG), sociology and linguistics of medicine (CK), medical anthropology (AN), and caregiving, primary care, and health services research (VY). All 4 were investigators at the National VA Center for Innovation to Implementation based at the Palo Alto VA Health Care System. With the exception of VY, who worked with one of the national BBC leaders in a separate study 5 years before this study, the qualitative team did not have prior relationships with the study participants. The qualitative team codeveloped the semistructured interview guides based on PARIHS constructs and pilot tested them with 1 caregiver and 2 staff members at the Palo Alto VA Medical Center before finalizing them (Multimedia Appendix 3).

Data collection consisted of 45-min one-to-one phone-based interviews that were audio-recorded and transcribed by professional transcriptionists and field notes that were recorded by interviewers immediately following interview completion. Transcripts were not returned to the participants for comments or corrections. In caregiver interviews, we asked about caregiving responsibilities, experiences with the VA, use of online technology, and preferences for caregiver support, education, and skills training. Among caregivers who had not enrolled but were aware of the workshop, we asked about outreach or enrollment processes they had experienced, why they had not enrolled, and for any other feedback they chose to share. Among caregivers who had enrolled, we asked about reasons for enrollment and feedback on their outreach, enrollment, and workshop experiences, suggestions for improvements, and whether the workshop had impacted them. In interviews with local staff and regional and national leaders, we collected feedback corresponding to all PARIHS constructs, including asking about their role, training, activities, and observations during the BBC rollout and suggestions for improvements.

The qualitative team codeveloped the codebook. Deductive codes were based on PARIHS constructs, whereas inductive codes were developed using content analysis of transcripts of a subset of 10 interviews and accompanying field notes [29]. Following the development of the initial codebook, 3 coders (VY, RG, and AN) applied a rapid analysis matrix coding form to evaluate an additional 13 interviews, with 2 coders coding each interview (double-coding) [30]. The initial matrix coding form consisted of rows for participants, codes as column headers, and transcript quotes and notes in the pertinent cells displayed in an Excel spreadsheet (Microsoft Corp). The full qualitative evaluation team then met and reviewed the matrices, including codes, quotes, and notes. CK (noncoder) examined quotes and codes to ensure consistency and facilitated discussion of these (eg, of areas of agreement or contention or areas that required clarification), suggested modifications or additions to the codes, and elicited team agreement on the final coding form. Coders applied the final coding form to all interview data, including interviews previously coded at earlier stages. The full qualitative team met again to identify, define, and reach consensus on emergent themes. The team identified themes that addressed strengths and weaknesses of workshop implementation, similarities, and differences in participant feedback from low- and high-enrollment sites and possible strategies for optimizing uptake. Participants did not provide feedback on the findings.

### Integration of Survey and Interview Results

Next, we convened a half-day virtual meeting with our study team, advisory board, and a CSP representative using web-based conferencing capabilities. Attendees viewed survey and interview results displayed concurrently by the relevant PARIHS construct. Nonstudy team attendees (advisory board and CSP representative) commented on and confirmed the face validity of the results. All attendees then discussed and interpreted the results and reached a consensus on the integrated study findings.

### Identification of Strategies Likely to Improve Workshop Uptake

Building from integrated study findings, at the same meeting, attendees identified broad strategies likely to improve workshop uptake and agreed that these strategies should be recommended to VA BBC national leaders. The study team reported both integrated study findings and recommendations to national leaders in the form of a written report and webinar meetings.

### Stage 2: Determination Whether New or Enhanced Implementation Strategies Were Adopted and Whether Workshop Enrollment Changed

In 2018, we performed a follow-up evaluation consisting of interviews and longitudinal workshop enrollment data from the VA CSP operations databases to determine whether new or enhanced implementation strategies had been adopted and whether workshop enrollment changed.

### Follow-Up Interviews With National Leaders

In spring 2018, we conducted one-on-one phone interviews with 3 of the original 4 national leaders. The 4th leader was no longer working in the same position and was unavailable for the interview. Our goal was to learn about changes that had been made in response to the initial implementation evaluation. Questions focused on whether existing implementation strategies (from the 2013-2014 timeframe) had been enhanced and new strategies had been adopted (eg, based on stage 1 recommendations) and whether the workshop itself had been maintained. We used the same codebook established from the original 2015 interviews to analyze data from the follow-up interviews.

### Longitudinal Data on Workshop Enrollment

Following interviews, we used VA CSP operations data to retrospectively evaluate longitudinal workshop enrollment for the VA as a whole from 2013 to 2018. Data were available by VA fiscal year (FY; October 1 to September 30 of the following year). FY data correspond to the following periods of interest in this study:

1. 2013-2014: BBC workshop rolled out nationally.
2. 2015: study team performed initial implementation evaluation.
3. Late 2015 and early 2016: VA adopted expanded implementation strategies (as determined in follow-up interviews).
4. 2016-2018: follow-up period.

We graphically displayed the longitudinal data for visual inspection to determine the potential impact of expanded implementation strategies on workshop enrollment.

## Results

### Stage 1: Participants

Thirty-two caregivers, 13 local staff, and 9 regional and national leaders participated in surveys and interviews (Table 2). Approximately half of the caregivers (15/32, 47%) and local staff (7/13, 54%) were from low-enrollment centers. A total of 17 (53%) caregivers supported younger veterans from the post-9/11 era of service. All cared for veterans at high risk for morbidity and mortality based on their health conditions. Most were White, female, above the age of 40 years, and married to their care partners. Their care partner relationships (eg, spouse, child) were similar in distribution to those reported by caregivers enrolled in BBC during the first 2 years of workshop rollout (Multimedia Appendix 4). The chronic conditions of their care partners were also broadly similar to those of caregivers enrolled in BBC (Multimedia Appendix 4). Among the local staff, 69% (9/13) were caregiver support coordinators. Among regional and national leaders, 78% (7/9) were VA based and 2 (22%) were from the national organization responsible for administering the workshops on behalf of the VA.

**Table 2:** Characteristics of caregivers, local staff, and regional and national leaders.

Participant group and characteristics			Participants, n (%)
**Caregivers (n=32)**			
	**Gender**		
		Female	28 (88)
		Male	4 (13)
	**Age by category (years)**		
		18-29	4 (13)
		30-39	3 (9)
		40-49	6 (19)
		50-59	6 (19)
		60 and older	4 (13)
		Not stated	9 (28)
	**Race/ethnicity**		
		White	20 (63)
		Other^a^	3 (9)
		Not stated	9 (28)
	**Relationship with veteran**		
		Spouse	26 (81)
		Child	3 (9)
		Sibling	2 (6)
		Parent	1 (3)
	**Number of chronic conditions for which providing support**		
		3 or more conditions	26 (81)
		1-2 conditions^b^	6 (19)
	**Experience with workshop**		
		Enrolled	14 (44)
		Aware of it but not enrolled	10 (33)
		Not aware	8 (25)
**Local staff (n=13)**			
	Social worker in role of caregiver support coordinator		9 (69)
	Other staff^c^		4 (31)
**Leaders (n=9)**			
	VA^d^ regional position		5 (56)
	VA national position		2 (22)
	National organization running workshops		2 (22)

^a^Hispanic/Latino (n=1), Native American/Alaskan Native (n=1), and Other (n=1).

^b^All of the conditions are associated with high morbidity and mortality and include traumatic brain injury, posttraumatic stress disorder, schizophrenia, dementia, and chronic obstructive pulmonary disease.

^c^Other staff include 3 primary care social workers and 1 clinical psychologist.

^d^VA: US Department of Veterans Affairs.

### Survey Results

A total of 72% (23/32) of caregivers completed the surveys. They reported high rates of increased stress (21/23, 91%), depression symptoms (19/23, 83%), sleep deprivation (19/23, 83%), neglect of healthy physical activity (15/23, 64%), neglect of healthy eating habits (14/23, 61%), and delay of own health care (11/23, 48%) since becoming a caregiver, which are comparable with national data on veteran caregivers [20]. Among caregivers enrolled in the workshop, 71% (10/14) completed surveys. In response to questions that correspond to the PARIHS evidence construct, 100% (10/10) reported that they would recommend the workshop to others and that their quality of life had improved (8/10, 80%) or may have improved (2/10, 20%) as a result of what they had learned in the workshop.

Among the local staff, 10 (77%) completed surveys: 71% (5/7) from low-enrollment centers and 83% (5/6) from high-enrollment centers. Describing whether workshop implementation at their center had been a success, all 5 staff members from high-enrollment centers agreed, whereas none of the 5 from low-enrollment centers agreed. Nonetheless, most felt that the BBC workshop had been accepted by caregivers and was supported by research and that they had received useful informational materials for caregivers and educational materials for themselves (Table 3). In contrast, a minority reported having a regional mentor who could assist them or having a system for tracking caregivers.

**Table 3:** Local staff survey results according to Promoting Action on Research Implementation in Health Services constructs.

PARIHS^a^ construct	Survey question	Staff in agreement with statement (n=10), n (%)	Linkages to Table 4 theme and whether survey responses are consistent with Table 4 theme^b^
1. Evidence—caregiver experiences	BBC^c^ has been well-accepted by VA^d^ caregivers	7 (70)	Theme 2a: Yes^e^
2. Evidence—research evidence	BBC is supported by research evidence	7 (70)	Theme 2b: Yes
3. Context—staff resources	Informational materials are available for caregivers and staff to raise awareness about the workshop	7 (70)	Theme 3b: Yes^f^ Theme 4d: No^f^
4. Facilitation—staff skills and attributes	Resources to educate staff about workshop structure and content are available	7 (70)	Theme 5a: Partial^g^
5. Facilitation—availability of external facilitator to mentor and assist staff	A regional mentor who can help answer questions or solve problems is available	3 (30)	Theme 5a: Yes
6. Context—information technology capabilities	An approach used at our facility to evaluate and improve implementation includes a system of tracking which caregivers have been referred to BBC	4 (40)	Theme 5b: Yes

^a^PARIHS: Promoting Action on Research Implementation in Health Services.

^b^Linkages (including areas of consistency and inconsistency) between quantitative and qualitative results were discussed during our process of integrating stage 1 findings, as described in the Methods section.

^c^BBC: Building Better Caregivers.

^d^VA: US Department of Veterans Affairs.

^e^Caregiver responses to survey questions on the PARIHS evidence construct are described in the text and are consistent with these staff responses, as well as with Theme 2a.

^f^Explanation for both a *yes* and a *no* designation: qualitative results in the text and Table 4 indicate that certain outreach materials, particularly those useful for post-9/11 caregivers, were readily available (and used by staff) during workshop rollout, which is consistent with Theme 3b. However, outreach materials, mechanisms, and contacts for pre-9/11 caregivers and others were insufficient (as summarized by Theme 4d), which explains why this survey finding was felt to be inconsistent with Theme 4d.

^g^Explanation for *partial* designation: qualitative results in the text and Table 4, Theme 5a, explicitly recognize that training was insufficient for many new staff members but, conversely, they also imply that experienced staff (present during the early rollout) were generally satisfied with their training, which explains why this survey finding was determined to be partially consistent with Theme 5a.

**Table 4:** Qualitative themes on workshop implementation: overarching theme and themes on strengths and weaknesses.

Theme and subtheme			Exemplar quotes	PARIHS^a^ construct	Linkages to Table 3 rows
**Overarching theme**					
	**1. Importance of outreach and marketing to prompt workshop enrollment**		Local staff, low-enrollment center: “If it was more advertised and marketed, I think that would be good.” Regional leader: “I feel like it’s an easy sell. We have the promotional materials to send out.” National leader: “I’ve learned that it’s important to have marketing materials for caregivers. That’s a big lesson.”	Spans all PARIHS constructs and implementation strengths and weaknesses	N/A^b^
**Implementation strengths**					
	**2. Belief in positive impact of workshop encouraged uptake**				
		2a. Positive caregiver experiences during rollout	Caregiver: “You learned a lot of really good tools that I use in my daily life. And you could also communicate with the other class members.” Local staff, high-enrollment center: “When the caregivers took the course, I got such positive feedback from them, it made me a believer.” Regional leader: “Anybody that has participated in it has really given lots of positive feedback. And probably the most telling thing is the fact that staff continue to make the referrals.”	Evidence—caregiver experiences are positive	Row 1
		2b. Value of evidence from prior research^c^	Local staff, low-enrollment center: “You want something that's, you know, evidence-based.” Regional leader: “The research definitely does matter. Because like I said, you're pitching an additional task to [caregivers] who are super busy.”	Evidence—research evidence is convincing	Row 2
	**3. Successful outreach to some caregiver groups**				
		3a. Use of stories and testimonials from trusted sources	Caregiver: “[Caregivers will enroll] if they have good caregiver coordinators that push the program and say, “This is something that you really need to do.” Or the therapists, whoever they trust.” Local staff, high-enrollment center: “My suggestion [for other centers] would be short little testimonials of people who've taken the course and it would say, ‘See what Mary said about the course.’” Regional leader: “I always tell people when promoting it—caregivers when they link up they’ve really enjoyed it.”	Evidence—materials help caregiver determine that program is likely to meet their needs; Facilitation—staff have necessary skills	N/A
		3b. Multiple contact episodes and materials	Caregiver: “First I heard verbally about it. Then they sent me a flier regarding it and said, ‘Here is what it is – read it and see if this is what you're looking for.’ Then he followed up with a phone call. I think the more information, the better.” Local staff, low-enrollment center: “I would say the biggest thing [hindering enrollment] is that we don’t have a follow-up plan—a reminder. Because sometimes that reminder helps. To talk about it and you know, give them a little push.” Regional leader: “We’ve sent out like a large volume of fliers—and I’ve pitched it to people in person and over the phone and things like that. You can't just do it once. You have to repeatedly send stuff out or bring it up.”	Context—staff have sufficient resources; Facilitation—staff have necessary skills	Row 3
**Implementation weaknesses and the needs they suggest**					
	**4. Missed opportunities for improved outreach**				
		4a. Detailed information on workshop content and structure	Caregiver—who knew about the workshop but had not enrolled: “Provide more actual information on the content, not just a link on the computer.” Local staff, low-enrollment center: “I don't see how the system works. I would like that. One caregiver was telling me that she didn't feel there was enough information, but without seeing it, I couldn't respond to her.” Regional leader: “Some of the CSCs^d^ do not understand the details. I think it would be helpful to train the coordinators on what actually is in [it]. Because—marketing wise—you're not going to refer people as readily to something that you don't have knowledge about.”	Evidence—caregiver cannot determine if program is likely to meet their needs; Evidence—staff cannot observe program	N/A
		4b. Expanded online mechanisms for outreach and enrollment	Local staff, low-enrollment center: “My suggestion would be a link on the site [portal to electronic health records]—on the same page, if caregivers could just click on right there.” Local staff, high-enrollment center: “If the general caregivers [caregivers of older, pre-9/11 era veterans] could access it from the caregiver website, that might be really good.” National leader: “There's a barrier in that you have to pick up the phone and find your CSC. So the recruitment process is interrupted by the fact that it's not a complete online experience, even though the rest of their experience will be online.”	Context—lack of technology tools	N/A
		4c. Partnership with communities and community groups	Only 1 caregiver learned about the workshop through a community group: “Crossroads [VA-funded nonprofit] is where he goes once a month. They just said, ‘Well, try this and maybe they can help you.’ So I picked up a brochure.”^e^ Only 1 local staff member, at a high-enrollment center, described doing outreach to the community: “I do community outreach events where I put out that fact sheet.”^e^ National leader: “We have tremendous access to community-based organizations that are working with caregivers all over the country. And we've wanted to promote the program through that [but have not been able to].”	Context—limited awareness among external networks and communities	N/A
		4d. Increased outreach to certain caregiver groups and their health care teams	Pre-9/11 era caregiver: “I don’t think it’s widely known that you have this. None of the doctors told me about it.” Local staff, low-enrollment center: “I definitely think outreach and mailers could be made more regularly to the general [pre-9/11 era] caregivers. It just kind of goes by the wayside.” National leader: “One of the goals is to increase the number of general [pre-9/11 era] caregivers in the program. I don't think the coordinators have as much contact with them.”	Context—limited awareness among some stakeholder groups; Context—limited staff resources and time	Row 3
	**5. Missed opportunities to support staff in outreach efforts**				
		5a. Training and mentoring for new staff	Local staff hired after the rollout began, low-enrollment center: “I had no one to collaborate with, no one to talk to. I think new CSCs need to be more knowledgeable of the program.” Regional leader: “The initial rollout was either really early in my assuming responsibility for this role or shortly thereafter. Initially it flew right past me.”	Facilitation—lack of training and mentoring from experts	Rows 4 and 5
		5b. Improved data management capabilities to Generate outreach contacts Track caregivers Target follow-up outreach	Local staff, low-enrollment center: “A quarterly flier might just remind people of the opportunity to participate in this program. At the national level can they generate mailers?” Local staff, high-enrollment center: “It would be very beneficial to have a way to pull up the list of people that we have referred that have never taken any steps forward, so that we can hit a button and they get a reminder email.” Regional leader: “The website where you can see where the caregiver is in the process, I don't always check it. It probably would be helpful. But you don't always have the time to check individually.” Regional leader: “Once we make referrals, we’re not involved. Unless there was some sort of process where if the caregiver didn’t follow through, [workshop organizers] came back to the coordinator and said, ‘Will you see if they're still interested?’ There’s no mechanism for that.”	Context—lack of information technology capabilities	Row 6

^a^PARIHS: Promoting Action on Research Implementation in Health Services.

^b^N/A: not applicable.

^c^Caregiver participants did not make comments about research evidence.

^d^CSC: caregiver support coordinator.

^e^These are not exemplar quotes. They are the only cases in which respondents described using these outreach mechanisms and thus highlight their relative absence from use.

### Interview Results

During interviews, participants from low-enrollment centers identified fewer implementation strengths and a greater number of weaknesses than those from high-enrollment centers. However, both groups generated similar implementation themes. As the analyses identified common themes, we report findings from low- and high-enrollment centers together.

#### Theme 1: Importance of Outreach and Marketing to Prompt Workshop Enrollment

We identified an overarching, general theme on the need for effective outreach and marketing to promote workshop enrollment and thereby implementation success (Table 4, theme 1). This theme emerged from comments on implementation strengths and weaknesses and spanned all PARIHS constructs. All participant groups (caregivers, staff, and leaders) emphasized this need:

They need to spread the information. Blast it out. Just get the word out.

Caregiver

In addition, staff and leaders used marketing language (eg, “market,” “advertise,” “sell,” “promote”) to describe characteristics of the rollout, even in cases where they were not reaching as many caregivers as they would have liked. Of note, none of our survey questions were designed to elicit input on outreach and marketing efforts other than the questions for staff on *informational materials*, as described above.

Respondent feedback also generated themes on specific implementation strengths and weaknesses. Identified themes map onto the PARIHS constructs of evidence, context, and facilitation. They include 2 themes on implementation strengths and 2 on implementation weaknesses, each with respective subthemes.

#### Theme 2: Belief in Positive Impact of Workshop Encouraged Uptake

All participant groups described an overall positive impact of the workshop. In doing so, they commented on the experiences, needs, and preferences of caregivers as well as research evidence. Both forms of *evidence* were highlighted as important to successful implementation because they made the workshop attractive (to caregivers and staff) and easier to promote.

##### Subtheme: Positive Caregiver Experiences During Rollout

Caregivers, staff, and leaders identified the workshop as a positive experience for most caregivers (Table 4, subtheme 2a). Caregivers reported that it met expectations of improving their skills and social support, and staff reported receiving similar feedback directly from their local caregivers:

In the hallways, they'll stop me saying, “I've been using that online support group, it's really good.”

Local staff, low-enrollment center

All groups valued such feedback as evidence of workshop effectiveness.

##### Subtheme: Evidence From Prior Research

Local staff and regional and national leaders expressed that they also valued research evidence as a motivating factor for their implementation efforts (Table 4, subtheme 2b):

Research support matters because it's a waste of time if it's not proven to be effective. And the last thing we want to do is waste caregivers' very precious time.

Local staff, high-enrollment center

Research evidence assured them that the workshop was worthwhile for busy caregivers. Caregivers did not mention research evidence in their comments.

#### Theme 3: Successful Outreach Consisted of Trusted Stories and Multiple Contacts

Another noted strength of the rollout was the achievement of effective outreach to some caregiver groups—specifically, caregivers of younger veterans from the post-9/11 era. These caregivers were best known to local caregiver support coordinators because an act of Congress in 2010 enabled many of them to receive a caregiving stipend from the VA, but payment required quarterly contact with coordinators. In contrast, caregivers of older, pre-9/11 era veterans were not eligible for the stipend, making them less likely to come to the attention of coordinators. Among the post-9/11 caregivers for whom outreach efforts were more successful, certain strategies were identified as important to that success.

##### Subtheme: Use of Stories and Testimonials From Trusted Sources

Comments from caregivers, local staff, and leaders highlighted that outreach was more effective if it involved caregiver stories and testimonials from trusted sources (Table 4, subtheme 3a):

The person who I talked to gave me all the caregiver feedback about its friendly atmosphere. It was a good fit.

Caregiver

This type of information was characterized as trustworthy and relatable for caregivers. Knowing that the workshop was helpful to other caregivers prompted them to try it for themselves.

##### Subtheme: Use of Multiple Contact Episodes and Materials

Caregivers, staff, and leaders described successful outreach as requiring multiple outreach contacts (*touches*) and a variety of formats, for example, in person, fliers, emails, and phone calls (Table 4, subtheme 3b):

They teach us in sales that sometimes it takes quite a few touches to convince somebody to even take a look at what you’re trying to sell them. We’re trying to sell them on the idea of doing this course.

Local staff, high-enrollment center

These repeated contacts reminded caregivers that they could enroll in the workshop when they were at a place in their busy lives to be ready and able to do so.

#### Theme 4: Missed Opportunities for Improved Outreach Limited Workshop Uptake

Caregivers, local staff, and leaders also identified a number of implementation weaknesses. They described missed opportunities for providing effective outreach to caregivers overall and to particular caregiver subgroups, such as caregivers of older, pre-9/11 era veterans, and suggested the need for implementation changes described in the subthemes below.

##### Subtheme: Detailed Information on Workshop Content and Structure

Some caregivers felt that a lack of detailed information about the workshop on outreach materials inhibited their interest in the program (Table 4, subtheme 4a). Local staff recognized a parallel issue—that their lack of detailed information on the workshop or opportunity to observe it for themselves limited their understanding of it, which constrained their ability to market it:

It would be great if we could actually see the information that the caregivers are receiving. Because we could give more feedback on it to caregivers that way—on the modules and what it all entails.

Local staff, high-enrollment center

Regional leaders had similar insights on the need to improve staff familiarity with the workshop.

##### Subtheme: Expanded Online Mechanisms for Outreach and Enrollment

Given that the workshop was web-based, many caregivers were confused by the minimal amount of online information about it and the absence of an online enrollment mechanism. They found these to be barriers to enrollment:

When I go on the VA website, there’s not too much on the web about it. You got to call this number to find out who the coordinator is, then get in contact. I mean it’s so much work to get there.

Caregiver

Local staff also desired online options for caregivers to receive information and enroll, particularly caregivers with whom they had little ongoing contact (eg, pre-9/11 era caregivers; Table 4, subtheme 4b). Some leaders agreed that the lack of online mechanisms for outreach and enrollment was an implementation weakness.

##### Subtheme: Partnership With Communities and Community Groups

Caregivers reported participating in or seeking support from a variety of community groups and community networking sites, many of which were well-established at local, regional, and national levels, such as Veterans of Foreign Wars, Hearts of Valor, the Wounded Warriors Project, Operation Homefront, and Facebook groups for veteran wives. However, there is little evidence to suggest that these community groups were engaged as partners in workshop outreach efforts. Only 1 caregiver reported receiving information on the workshop from a community organization and, in this case, it was a VA-funded nonprofit (Table 4, subtheme 4c). Other caregivers reported hearing about the workshop by word of mouth from veteran spouses who had completed the workshop. Caregivers identified the lack of effective outreach in the community as a missed opportunity:

I didn’t even know it existed. I had to find it out through somebody I work with. People should know that this program exists and not just stumble upon it.

Caregiver

Similarly, among local staff and regional leaders, only 1 person described performing outreach to community groups. Some leaders recognized community outreach as an important component missing from existing outreach efforts.

##### Subtheme: Increased Outreach to Certain Caregiver Groups and Their Health Care Teams

Caregivers of older, pre-9/11 veterans expressed insight into the limited outreach efforts made to reach them (Table 4, subtheme 4d). Local staff and leaders made parallel comments about the need to improve targeting of these caregivers as well as other caregiver subgroups (eg, male caregivers of female veterans). They suggested working with health care teams and other support services that commonly interact with older veterans and their caregivers—for example, those in primary care, home health, mental health, respite care, physical and occupational therapy, transportation, and social work outside of the CSP:

Partner with the home-based primary care, community-based care, and the PACT [patient-centered medical home] teams. Because that’s where you are going to get your older population.

Regional leader

#### Theme 5: Missed Opportunities to Support Staff in Outreach Efforts

Many staff and some regional leaders reported that they did not have the support necessary to perform or maintain effective workshop outreach and indicated the need for improvements described in the subthemes below.

##### Subtheme: Training and Mentoring for New Staff

Staff who attended the original webinar training on the workshop and rollout gave overall positive feedback (although many still desired to observe the workshop itself, as described above). However, some new staff who began as coordinators after the initial training reported a lack of knowledge, skills, and mentoring. This undermined their motivation and ability to promote the workshop (Table 4, subtheme 5a). Even experienced staff observed this as a problem:

I think another national training needs to happen, so that the newer coordinators will see it. Because then I think that they would utilize it.

Local staff, high-enrollment center

Leaders also recognized the need for new staff to receive timely and thorough training and mentoring.

##### Subtheme: Improved Data Management Capabilities

Staff and regional leaders reported having inadequate data management capabilities for many outreach-related activities (Table 4, subtheme 5b). They described ad hoc, inefficient, or nonexistent processes:

I think we did have an internal spreadsheet going at one time, but I don’t think we’ve followed up with that.

Local staff, low-enrollment center

Staff desired efficient approaches to data processing, especially if they could be performed at the level of the national VA *to generate regular, multiple outreach contacts* to caregivers at their local centers. Second, staff and regional leaders noted that they lacked standardized tools *to track caregivers* along the outreach-to-enrollment pathway. Some staff improvised their own local tracking tools but had trouble maintaining them. Third, the national workshop enrollment database was not integrated with local databases, making it time-consuming to access and infrequently used. These poor data management capabilities undermined the ability of staff *to target follow-up outreach* to specific caregivers. Although local staff felt these deficiencies most acutely, regional leaders also recognized them.

#### Divergent and Lone Comments

We identified divergent comments from caregivers and staff. Two caregivers expressed that their workshop experience would have been improved by having the other caregivers in their workshop be more like them, according to certain characteristics. One desired to be in a group with younger caregivers, whereas the other wanted to be in a group with caregivers whose care partners had the same medical condition as her care partner. Both recognized beneficial aspects of the existing workshop. A local staff member was supportive of the web-based workshop as a valuable option for caregivers but felt that in-person programs were more beneficial if caregivers could attend them. Finally, another staff member desired to have a Spanish workshop for Spanish-speaking caregivers.

### Integrated Findings on Implementation Strengths and Weaknesses

Our study team, advisory board, and a representative from VA CSP leadership determined that the survey and interview findings were consistent with a number of implementation strengths—specifically, that caregiver experiences with the workshop were positive, research evidence supported its use, and staff received some useful outreach materials and resources that helped them support the rollout (Table 3, last column, and Table 4, last column). Findings also converged on implementation weaknesses—that outreach materials and approaches were insufficient in multiple areas, certain staff and regional leaders did not receive necessary training or mentoring, and that data management capabilities were inconsistent or lacking. When we noted areas of apparent inconsistency between quantitative and qualitative results (Table 3, rows 3 and 4), we discussed and noted potential explanations (Table 3, footnotes f and g).

### Recommended Strategies for Improving Workshop Uptake

On the basis of the integrated findings, we identified multiple strategies likely to improve workshop uptake and recommended them to VA leadership in the form of a report. The strategies included investment in robust outreach and marketing capabilities, tailoring outreach strategies to all key stakeholder groups and subgroups, use of stories, testimonials, repetition, details, and diverse delivery options in marketing campaigns, recurrent training sessions and mentoring for new staff, and comprehensive data management and reporting capabilities (Table 5).

**Table 5:** Strategies identified as likely to improve workshop uptake and whether US Department of Veterans Affairs adopted them.

Strategies	Further details	Whether VA^a^ adopted
Invest in outreach and marketing capabilities	May need to contract out	Yes
Tailor specific outreach strategies to all key stakeholder groups	Groups include the following: Potential workshop participants—may require identifying and targeting important subgroups of caregivers Local implementation staff with primary responsibility for implementing workshop (ie, caregiver support coordinators) Other staff who interact with caregivers or their care partners Community groups	Partially
Use campaigns that are personal, repeated, include workshop details, and have diverse delivery options	Use personal stories and testimonials—from peers (ie, caregiver peers and staff peers) Repeat outreach contacts over time—with all key groups, not just caregivers Include adequate information on workshop details Enable diverse delivery mechanisms—both electronic and nonelectronic	Yes
Conduct recurrent training and mentoring for new staff	Is especially important for new staff at locations with no other experienced staff on-site	Yes
Perform comprehensive, ongoing data management and reporting	To support and track outreach efforts, enrollments, and follow-up on unsuccessful enrollments	Partially

^a^VA: US Department of Veterans Affairs.

**Stage 2: Findings on New and Enhanced Implementation Strategies**

During follow-up interviews in 2018, VA leaders identified multiple implementation changes made in response to stage 1 findings and recommendations (Table 5, last column; see Multimedia Appendix 5 for additional details). The VA CSP contracted with a new organization with greater marketing expertise to administer the workshop, and together they developed and adopted both new and enhanced outreach approaches. They tailored new marketing materials and outreach to implementation staff (eg, new monthly newsletters for caregiver support coordinators), some VA nonimplementation staff and services in close contact with pre-9/11 veterans and their caregivers (eg, primary care social work, home health nursing), and some veteran service organizations and communities (eg, through presentations and press releases). They highlighted stories for caregivers in multiple new ways (eg, videos, quotes, photographs) and did the same for implementation staff (eg, caregiver support coordinators sharing their *BBC stories* with each other in videos or listservs). Outreach campaigns incorporated more repeat *touches* with both groups (eg, regular email invitations to caregivers), diverse forms of delivery (eg, social media posts and phone-based blurbs) and more detailed information on workshop structure and content. For instance, leadership approved and built a demonstration workshop so that staff could learn about workshop details firsthand and then pass the information to the caregivers with whom they worked. Expanded materials for new staff include recurring national training and reminders about how to access outreach resources. Finally, the workshop platform began regularly to email staff with reports on the enrollment status of caregivers at their respective medical centers.

Leaders also reported that they were unable to address some implementation weaknesses. The CSP website, which was managed by a different VA entity with limited funding, was not updated to include more information on the workshop and links to it. The VA national database of registered caregivers remained unlinked from the workshop database, and so it could not be used to generate workshop outreach invitations or other communications to caregivers. Finally, there was no plan to develop a workshop in Spanish.

### Longitudinal Workshop Enrollment

**Figure 1:**
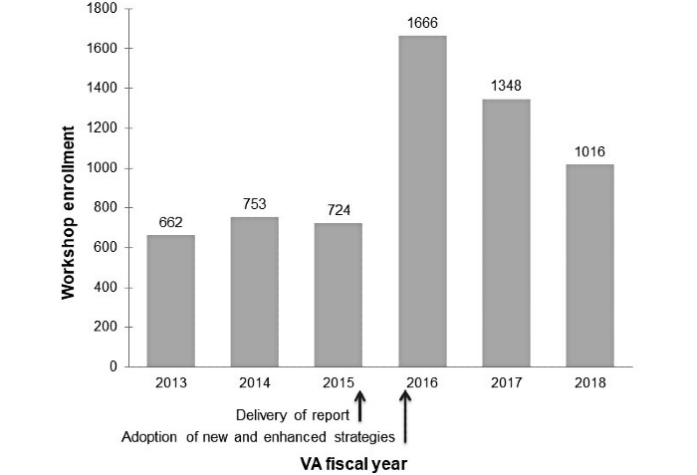
Workshop enrollment, 2013-2018. US Department of VA fiscal years encompass October through the end of September the following year. The study team reported integrated study findings and recommended new and enhanced strategies likely to improve workshop uptake to VA Building Better Caregivers national leaders in the late fiscal year 2015. In late 2015 and early 2016, the VA adopted a number of these recommended implementation strategies. VA: Veterans Affairs.

Visual inspection of longitudinal enrollment data indicates a possible impact of the new and enhanced implementation strategies. During the initial BBC rollout period (2013 to 2014) and the implementation evaluation period (2015), annual enrollment was approximately 700 caregivers per VA FY (Figure 1). Following implementation changes made in late FY 2015 and early FY 2016, annual workshop enrollment increased. In FYs 2013 to 2015, cumulative enrollment was 2139, which rose to 4030 in FYs 2016 to 2018. Over 6 years, cumulative BBC enrollment reached 6169, which equates to 12% of approximately 50,000 caregivers registered with the VA.

## Discussion

### Principal Findings

This study expands the limited implementation science literature on best practices for health systems to use when implementing web-based psychoeducational programs [13,31]. In our evaluation of the BBC workshop rollout among VA centers nationwide, we identified implementation strengths and weaknesses, including belief in the positive impact of the workshop, use of some successful outreach approaches for caregivers, and missed opportunities to improve outreach and to support both new and experienced implementation staff. On the basis of these findings, we identified and recommended to VA leadership new or enhanced implementation strategies likely to improve workshop uptake—specifically, increased investment in outreach and marketing capabilities, tailoring outreach strategies to key stakeholder groups and subgroups, use of campaigns that are personal, repeated, detailed, and have diverse delivery options, recurrent training and mentoring for new staff, and comprehensive data management and reporting capabilities to support staff implementation efforts. We determined that the VA adopted many of these strategies and, in the years following adoption, national BBC enrollment increased.

### Limitations

Our evaluation has a number of limitations. We initiated this after the workshop rollout was underway, rather than prospectively. At the same time, this enabled us to quickly identify low- and high-enrollment sites for inclusion in the evaluation. Our evaluation is also limited to a single health care system. Nonetheless, the VA health care system is the largest integrated health care system in the United States and, as such, it has numerous medical centers. These demonstrated variation in implementation success as well as geography and catchment size, which enhanced the robustness of our evaluation. The VA workshop is available only in English, which precludes use by caregivers who have limited English proficiency.

### Comparison With Prior Work

Among caregiver programs, many have been funded as research projects but few, if any, have been the focus of published implementation evaluations. A recent systematic review identified 17 studies of web-based caregiver programs focused on education, health, and well-being [7]. However, we were unable to identify any with a published implementation evaluation. Among web-based caregiver programs identified by other means [32, 33], no implementation evaluations were found. Finally, we identified a process evaluation of a web-based intervention to support frail older adults that, at their discretion, could also involve their caregivers but was not designed for caregivers [34]. Thus, it remains to be seen how results from the current implementation evaluation may compare with findings from implementation evaluations of other web-based psychoeducational programs for caregivers.

Among caregivers who care for military veterans, US VA caregivers may be unique in the amount of support they receive. Since 2010, the VA and VA CSP have received substantial federal funding for caregiver programs and research. This has enabled the US VA and VA investigators to develop the BBC workshop and other VA caregiver programs (eg, Resources for Enhancing All Caregivers Health [REACH] VA); assess such programs for efficacy; fund their implementation; and in the case of this study, evaluate their implementation [22,35]. We are unaware of funding opportunities on the same scale in other countries. This may explain why evidence on the efficacy and implementation of veteran caregiver programs outside the United States is limited. In an environmental scan of resources (defined as programs and services) for families of veterans with operational stress injuries, investigators identified 42 non–United States resources but concluded, “Information on evaluation and evidence for resources is limited*”* [36]. To our knowledge, none were the focus of an implementation evaluation.

If one examines web-based or technology-based interventions for patients rather than caregivers, implementation data remain sparse [4,31,34,37,38]. A 2013 systematic review of studies of web-based patient self-management workshops determined that 87% “did not describe any measures taken to sustain the tested programs past designated study time periods” [31]. The remainder reported on the number of participants reached but not the strategies that were successful or unsuccessful in achieving participation. Not surprisingly, a similar 2013 review of web-based interventions for psychosocial health determined that issues of reach and adoption of web-based interventions were a crucial area for further research [4]. Four years later, a 2017 review of 268 randomized controlled trials described a proliferation of evidence-based self-guided internet health programs but concluded, “Unfortunately, most of the Internet delivered health interventions that were efficacious through RCTs were not available after the conclusion of the trials” [3] and we could not identify implementation evaluations of these same trials. Finally, in the case of eHealth services broadly defined (eg, telehealth, patient portals, web-based decision aids), others have identified a taxonomy of implementation barriers and facilitators from multiple studies and expert input [11], but there are few follow-up studies to determine whether making implementation enhancements suggested by the taxonomy does indeed boost uptake. Thus, experts have highlighted implementation research as the crucial next step in spreading the benefits of evidence-based digital health interventions to all those who might benefit [39].

This evaluation suggests that implementation research can help boost uptake of web-based psychoeducational interventions for caregivers and perhaps others. Among the results, we were most surprised by the prominence of findings related to outreach and marketing. The implementation science literature has not emphasized these concepts to any great extent, although this may be changing. This evaluation was performed before the publication of the most recent iteration of the PARIHS implementation framework known as the i-PARIHS framework [40]. i-PARIHS adds a new construct, “recipients” to existing constructs (evidence, context, and facilitation) and defines it as “the people who are affected by and influence implementation [of the intervention] at both the individual and collective team level” [40]. In this study, we found implementation could be strengthened when specific groups and subgroups of *recipients* (eg, caregivers, staff, community organizations) were explicitly identified for tailored outreach campaigns, which aligns with this new PARIHS construct. Implementation scientists have started to note that marketplace principles and storytelling may contribute to implementation success [41,42]. Similarly, the recent report on a US National Cancer Institute implementation accelerator program concluded that “researchers can benefit from learning the ‘language’ of business” when “trying to move their research from the lab to the real world” [43].

### Conclusions

In this mixed methods implementation evaluation of the web-based psychoeducational BBC workshop for US veteran caregivers, we identified initial implementation strengths and weaknesses as well as multiple strategies likely to improve workshop uptake—including strong investment in outreach and marketing capabilities, tailoring of specific outreach strategies to all key stakeholder groups, use of outreach campaigns that are personal, repeated, detailed, and have diverse delivery options, ongoing training and mentoring for new implementation staff, and comprehensive data management and reporting capabilities. Upon follow-up assessment, we determined that the VA enhanced or newly adopted a number of these recommended strategies nationally and that workshop enrollment subsequently increased. Other health systems may want to deploy these strategies when implementing their web-based programs.

## Appendix

Multimedia Appendix 1 Interview guides used for phone interviews of caregivers, staff, and leaders.

Multimedia Appendix 2 Caregiver paper-based survey.

Multimedia Appendix 3 Staff online survey.

Multimedia Appendix 4 Available US Department of Veterans Affairs operations data on characteristics of caregivers enrolled in BBC workshop during rollout years 1-2 and their veteran care partners.

Multimedia Appendix 5 Detailed description of implementation changes made by US Department of Veterans Affairs in response to stage 1 findings.

## Abbreviations

BBC: Building Better Caregivers
CSP: Caregiver Support Program
FY: fiscal year
ORCA: Organizational Readiness to Change Assessment
PARIHS: Promoting Action on Research Implementation in Health Services
VA: US Department of Veterans Affairs

## References

[1] Lorig K, Ritter PL, Plant K, Laurent DD, Kelly P, Rowe S (2013). The South Australia health chronic disease self-management internet trial. Health Educ Behav.

[2] Wantland DJ, Portillo CJ, Holzemer WL, Slaughter R, McGhee EM (2004). The effectiveness of web-based vs. non-web-based interventions: a meta-analysis of behavioral change outcomes. J Med Internet Res.

[3] Rogers MA, Lemmen K, Kramer R, Mann J, Chopra V (2017). Internet-delivered health interventions that work: systematic review of meta-analyses and evaluation of website availability. J Med Internet Res.

[4] Paul CL, Carey ML, Sanson-Fisher RW, Houlcroft LE, Turon HE (2013). The impact of web-based approaches on psychosocial health in chronic physical and mental health conditions. Health Educ Res.

[5] Heber E, Ebert DD, Lehr D, Cuijpers P, Berking M, Nobis S, Riper H (2017). The benefit of web- and computer-based interventions for stress: a systematic review and meta-analysis. J Med Internet Res.

[6] Zhou T, Li X, Pei Y, Gao J, Kong J (2016). Internet-based cognitive behavioural therapy for subthreshold depression: a systematic review and meta-analysis. BMC Psychiatry.

[7] Ploeg J, Markle-Reid M, Valaitis R, McAiney C, Duggleby W, Bartholomew A, Sherifali D (2017). Web-based interventions to improve mental health, general caregiving outcomes, and general health for informal caregivers of adults with chronic conditions living in the community: rapid evidence review. J Med Internet Res.

[8] Rost T, Stein J, Löbner M, Kersting A, Luck-Sikorski C, Riedel-Heller SG (2017). User acceptance of computerized cognitive behavioral therapy for depression: systematic review. J Med Internet Res.

[9] Coffey NT, Cassese J, Cai X, Garfinkel S, Patel D, Jones R, Shaewitz D, Weinstein AA (2017). Identifying and understanding the health information experiences and preferences of caregivers of individuals with either traumatic brain injury, spinal cord injury, or burn injury: a qualitative investigation. J Med Internet Res.

[10] Bennett GG, Glasgow RE (2009). The delivery of public health interventions via the internet: actualizing their potential. Annu Rev Public Health.

[11] Schreiweis B, Pobiruchin M, Strotbaum V, Suleder J, Wiesner M, Bergh B (2019). Barriers and facilitators to the implementation of eHealth services: systematic literature analysis. J Med Internet Res.

[12] Kohl LF, Crutzen R, de Vries NK (2013). Online prevention aimed at lifestyle behaviors: a systematic review of reviews. J Med Internet Res.

[13] Gitlin L, Marx K, Stanley I, Hodgson N (2015). Translating evidence-based dementia caregiving interventions into practice: state-of-the-science and next steps. Gerontologist.

[14] Teno JM, Price RA, Makaroun LK (2017). Challenges of measuring quality of community-based programs for seriously ill individuals and their families. Health Aff (Millwood).

[15] Schulz R, Beach SR, Friedman EM, Martsolf GR, Rodakowski J, James AE (2018). Changing structures and processes to support family caregivers of seriously ill patients. J Palliat Med.

[16] Ploeg J, Ali MU, Markle-Reid M, Valaitis R, Bartholomew A, Fitzpatrick-Lewis D, McAiney C, Sherifali D (2018). Caregiver-focused, web-based interventions: systematic review and meta-analysis (part 2). J Med Internet Res.

[17] Sherifali D, Ali MU, Ploeg J, Markle-Reid M, Valaitis R, Bartholomew A, Fitzpatrick-Lewis D, McAiney C (2018). Impact of internet-based interventions on caregiver mental health: systematic review and meta-analysis. J Med Internet Res.

[18] Barbabella F, Poli A, Andréasson F, Salzmann B, Papa R, Hanson E, Efthymiou A, Döhner H, Lancioni C, Civerchia P, Lamura G (2016). A web-based psychosocial intervention for family caregivers of older people: results from a mixed-methods study in three european countries. JMIR Res Protoc.

[19] Cheng S, Li K, Losada A, Zhang F, Au A, Thompson LW, Gallagher-Thompson D (2020). The effectiveness of nonpharmacological interventions for informal dementia caregivers: an updated systematic review and meta-analysis. Psychol Aging.

[20] (2010). Caregivers of Veterans – Serving on the Homefront. United Health Group.

[21] Ramchand R, Tanielian T, Fisher M, Vaughan C, Trail T, Epley C, Voorhies P, Robbins M, Robinson E, Ghosh-Dastidar B (2014). Hidden Heroes: America's Military Caregivers.

[22] Dupke NJ, Plant KL, Kosteas J (2016). Supporting caregivers of veterans online: a partnership of the national council on aging and VA. Fed Pract.

[23] Lorig K, Thompson-Gallagher D, Traylor L, Ritter PL, Laurent DD, Plant K, Thompson LW, Hahn TJ (2010). Building Better Caregivers: a pilot online support workshop for family caregivers of cognitively impaired adults. J Appl Gerontol.

[24] Lorig K, Ritter PL, Laurent DD, Yank V (2019). Building better caregivers: a pragmatic 12-month trial of a community-based workshop for caregivers of cognitively impaired adults. J Appl Gerontol.

[25] O'Cathain A, Murphy E, Nicholl J (2008). The quality of mixed methods studies in health services research. J Health Serv Res Policy.

[26] Rycroft-Malone J (2004). The PARIHS framework-a framework for guiding the implementation of evidence-based practice. J Nurs Care Qual.

[27] Stetler CB, Damschroder LJ, Helfrich CD, Hagedorn HJ (2011). A Guide for applying a revised version of the PARIHS framework for implementation. Implement Sci.

[28] Hagedorn HJ, Heideman PW (2010). The relationship between baseline organizational readiness to change assessment subscale scores and implementation of hepatitis prevention services in substance use disorders treatment clinics: a case study. Implement Sci.

[29] Thomas DR (2016). A general inductive approach for analyzing qualitative evaluation data. Am J Eval.

[30] Neal JW, Neal ZP, van Dyke E, Kornbluh M (2014). Expediting the analysis of qualitative data in evaluation. Am J Eval.

[31] Stellefson M, Chaney B, Barry AE, Chavarria E, Tennant B, Walsh-Childers K, Sriram P, Zagora J (2013). Web 2.0 chronic disease self-management for older adults: a systematic review. J Med Internet Res.

[32] Blusi M, Dalin R, Jong M (2014). The benefits of e-health support for older family caregivers in rural areas. J Telemed Telecare.

[33] Griffiths PC, Kovaleva M, Higgins M, Langston AH, Hepburn K (2018). Tele-savvy: an online program for dementia caregivers. Am J Alzheimers Dis Other Demen.

[34] Robben SH, Perry M, Huisjes M, van Nieuwenhuijzen L, Schers HJ, van Weel C, Rikkert MG, van Achterberg T, Heinen MM, Melis RJ (2012). Implementation of an innovative web-based conference table for community-dwelling frail older people, their informal caregivers and professionals: a process evaluation. BMC Health Serv Res.

[35] Nichols LO, Martindale-Adams J, Burns R, Graney MJ, Zuber J (2011). Translation of a dementia caregiver support program in a health care system-REACH VA. Arch Intern Med.

[36] Tam-Seto L, Cramm H, Norris D, Eichler M, Smith-Evans K (2016). An environmental scan of programs and services for families of veterans with operational stress injuries. Mil Behav Health.

[37] Hermes E, Burrone L, Perez E, Martino S, Rowe M (2018). Implementing internet-based self-care programs in primary care: qualitative analysis of determinants of practice for patients and providers. JMIR Ment Health.

[38] Wozney L, McGrath PJ, Gehring ND, Bennett K, Huguet A, Hartling L, Dyson MP, Soleimani A, Newton AS (2018). Emental healthcare technologies for anxiety and depression in childhood and adolescence: systematic review of studies reporting implementation outcomes. JMIR Ment Health.

[39] Buis L (2019). Implementation: the next giant hurdle to clinical transformation with digital health. J Med Internet Res.

[40] Harvey G, Kitson A (2016). PARIHS revisited: from heuristic to integrated framework for the successful implementation of knowledge into practice. Implement Sci.

[41] King DK, Shoup JA, Raebel MA, Anderson CB, Wagner NM, Ritzwoller DP, Bender BG (2020). Planning for implementation success using RE-AIM and CFIR frameworks: a qualitative study. Front Public Health.

[42] Brownson RC, Fielding JE, Green LW (2018). Building capacity for evidence-based public health: reconciling the pulls of practice and the push of research. Annu Rev Public Health.

[43] Oh A, Gaysynsky A, Knott C, Nock N, Erwin D, Vinson CA (2019). Customer discovery as a tool for moving behavioral interventions into the marketplace: insights from the NCI SPRINT program. Transl Behav Med.

