# Supplementary material for: Improving Uptake of a National Web-Based Psychoeducational Workshop for Informal Caregivers of Veterans: Mixed Methods Implementation Evaluation
Source: J Med Internet Res. 2021 Jan 7;23(1):e16495. doi: 10.2196/16495 (PMC7819783; doi:10.2196/16495)
Supplement: Multimedia Appendix 1 [file jmir_v23i1e16495_app1.doc]

**CAREGIVERS *-* SEMISTRUCTURED INTERVIEW GUIDE**

We are very interested in learning about your experiences as a caregiver and your role in providing care for a Veteran [family member/partner]. We hope that by talking with you we can learn from your experiences and assist the VA in developing programs and services to better meet the needs of caregivers like you and Veterans who need care/assistance. Just a few reminders before we get started. Feel free to ask questions as we go along. There are no right or wrong answers. If you feel uncomfortable with any questions or need to take a break, let me know. What we talk about today will be kept confidential. We'd like to audio record the interview to make sure we keep good track of our discussion today.

Do you have any questions before we get started?

Is it ok with you if I turn on the audio recorder? (If no, take notes)

- Yes
- No

**PART A: CAREGIVING CONTEXT**

*To get started, I’d like to learn more about you and your activities as a caregiver.*

1. Please tell me about your caregiving routine and responsibilities.
   1. Why does this person need care?
   2. What type of care do you provide?
   3. What challenges/problems have you experienced while caregiving?
   4. What aspects of caregiving do you find rewarding or satisfying?

**PART B: GENERAL VA SUPPORT FOR CAREGIVERS**

*Now that we've talked about your experiences as a caregiver, I'd like to know more about your experiences with the VA.*

Experience with VA

1. As a caregiver, how would you describe your experiences with the VA?
   1. Are there things you like/dislike about VA?
   2. How could the VA improve?
   3. What staff do you and your Veteran have the most contact with at the VA (e.g., social worker, physician, CSC)?
      1. Has anyone been helpful/unhelpful?
      2. Have you met the caregiver support coordinator?
      3. Who do you talk to about caregiver concerns/support?
   4. What additional caregiving resources or support do you need?

**PART C: EXPERIENCE WITH BBC PROGRAM**

*For this next part of the interview we'd like to learn more about your experiences participating/enrolling in the in the Building Better Caregivers program.*

BBC program context

1. Tell me about your experience with the Building Better Caregivers program.

[USE only 1 of the 2 following questions]

1. [*COMPLETED* the program:] How do you feel now that you’ve completed the program?
   1. Has the program impacted your own health or your Veteran’s health? If so, how?
2. [*DID NOT COMPLETE* the program:] We noticed you enrolled in the program but didn't complete the program. Can you tell me why that was?

Enrollment process

*I'd now like to learn about your first contact with the Building Better Caregivers program*

1. How did you learn about the program? (CSC, SW, Psych, physician)
   1. Did you seek support for caregiving or offered support program by someone?
2. How was the program described to you? (in person, flyer) seek support or offered support)
3. Why did you enroll in the program?

1. Walk me through the process of signing up for the BBC program (eligibility letter, ROI forms)
2. What could make signing up easier?
3. Were there any challenges to participating in the program?
   - Probe: logging in, navigating the program, completing sessions
   - How did you deal with these challenges?

Experience with Building Better Caregivers program

*Part of our job is to receive feedback from caregivers to improve the BBC program.*

1. What feedback can you give us on the program?
   - Most/least favorite features
2. Did the BBC program meet your expectations? Why/why not?
3. Are you using what you learned from the program in your life now? Why/why not?
4. You’ve told me a bit about the program. Do you have any specific comments about workshop topics, such as ones that worked well or ones that didn’t work well? [provide participant with the list below]
   - Stress-management
   - Action planning
   - Problem solving
   - Difficult care partner behaviors
   - Sleeping well
   - Healthy eating and fitness/exercise
   - Difficult emotions
   - Care partner emotions
   - Making decisions
   - Finding help
   - Communication
   - Working with health care system
   - Working with healthcare providers
   - Medication management
   - Making plans for the future

Suggestions for improvement

1. How could we improve the program?
   - What topics should be added to the program?
   - What could have been covered in more depth?
   - Are there certain types of caregivers you would prefer to participate with (for example, those caring for Veterans with the same conditions as your Veteran partner)?
2. The VA has faced some challenges enrolling caregivers in the BBC program.
   - How could we increase participation in BBC?
   - How could we make the program more appealing to caregivers?

BBC online format

*I’m interested in your thoughts about the web-based BBC format*

1. What was your experience with using the online BBC program?

- 1. How/where do you access the internet?
  2. What is your comfort level with using the internet/computer?
  3. Do you prefer an internet or in person caregiver program? Why?
  4. Do you have any concerns about privacy when using a computer based caregiver support program? Why or why not?

**SOCIAL SUPPORT**

Alumni Community

[Only ask next question of CG who *COMPLETED* program]

1. Have you participated in the online Alumni Community discussion group?
   - If so, tell me about this experience?
2. Do you talk about your caregiving concerns and experiences with other people (family, friends, caregivers, healthcare professionals)? Why/why not?
   - What do you talk about?
3. What do you think about attending support groups/meetings for caregivers?
4. What are some important topics to discuss with other caregivers about?
5. Would you prefer: in person meetings or internet based formats?

CONCLUSION

*Thanks so much for talking with me today. We really appreciate you taking the time to share your experiences and input.*

1. Is there anything else we should know about your experiences as a caregiver?

1. Is there anything we forgot to ask you about or that you would like to add to our discussion?

**LOCAL STAFF *–* SEMI-STRUCTURED INTERVIEW GUIDE**

The focus of this interview is to understand staff perceptions of and experiences with the Building Better Caregivers program in the VHA. By talking with you, we hope to learn how the program is working so we can understand what is working well and what might not be working well. Our overall goal is to understand the program to help it boost enrollment and improve caregivers’ experiences with it.

Just a few reminders before we get started. Our evaluation is not affiliated with the BBC program. We welcome any feedback. Feel free to ask questions as we go along. There are no right or wrong answers. If you feel uncomfortable with any questions or need to take a break, let me know. What we talk about today will be kept confidential and private. We'd like to audio record the interview to make sure we keep good track of our discussion today. Do you have any questions before we get started?

Is it ok with you if I turn on the audio recorder? (If no, take notes)

- Yes
- No

*If yes, begin recording. Be prepared to take notes should the participant refuse audio recording.*

**BBC Role**

*To start off, I’d like to learn about your role and experience with the Building Better Caregivers program.*

1. Tell me about your experience with the BBC program
   1. What is your role in the BBC program?
   2. Describe your role in referring caregivers to the program?
   3. How does your role with the BBC program fit/not fit with your other work responsibilities?
2. What do you think about your level of involvement with the program? (enough, too much, too little)
   1. What aspects of BBC take up more of your time? Less of your time?
   2. How might enrolling caregivers in the BBC impact your workload in the long run (e.g., some people mentioned enrolling caregivers can reduce workload)?

**Beliefs and Knowledge of BBC Program**

*I’d like to know more about how you first learned about the BBC program.*

1. How did you learn about the BBC program?
2. Why was the BBC program developed for the VA?
3. Does having research to support the BBC program matter to you? Why or why not?
4. What resources have you used to find out more information about the BBC program?
5. How does the BBC program compare to other programs that you offer to caregivers?

**Local BBC Processes**

*I’d now like learn more about the recruitment/referrals process to the BBC program at your VA.*

1. What is the process for informing other VA staff members about the BBC program?
   1. Who do you inform about the program?
   2. How do you describe the program to these staff members?
2. Walk me through the process of referring caregivers to the BBC program?
   1. How is a caregiver determined eligible for BBC?
   2. Who decides when to refer a caregiver?
   3. What is the eligibility for BBC?
   4. How is the program described to caregivers (in person, via phone, letter, how long does it take?)?
      1. What recruitment materials do you share with caregivers?
   5. Who is else is involved with this referral process? (other VA staff)
      1. What are their roles and responsibilities with BBC?
      2. Who is responsible for recruiting/enrolling caregivers into the BBC?
   6. Have you noticed things that have worked particularly well about your referral process?
   7. Have you noticed things that have not worked particularly well? What are they?
   8. In your opinion, how can the referral process be streamlined?
      1. How could that be implemented?
      2. What barriers do you anticipate in implementing that?
3. What are the referral and enrollment expectations for your site?
   1. How many? How often?
   2. What role does Central Office play in those expectations?

**Data Tracking & Evaluation**

*For these next few questions, I’m interested in learning if information is being collected about the BBC program and/or suggestions for collecting information about the BBC program.*

1. Is anyone tracking information about the BBC program?
   1. Why/why not?
2. What information is being collected (e.g., eligible, referred, enrolled, completed)?
3. Is this information being analyzed or evaluated?
   1. Why/why not?
4. Any other information that would be useful to have about the BBC program?
5. What is the best way to collect this information/data?
6. Who should be responsible for collecting this information?
7. How do they/should they keep track of this data? (database)
8. What happens to the data? (shared with others?)

**Challenges with BBC Program - Internal Facilitators**

*I would now like learn about any challenges you might be having with the BBC program*

1. What are some challenges you’ve had with the BBC program? (referrals, enrollment, staff, resources, recruitment)
2. Is there someone responsible for dealing with these challenges?
   - 1. If yes, who is this person?
     2. How have they dealt with these challenges?
     3. Has it been helpful?
3. Is anyone responsible for working with the local leadership on BBC-related challenges?
   - 1. If yes, what is his or her process for doing this?
        1. Has it been helpful?

**Enrollment Challenges**

1. Some VA sites are facing challenges enrolling caregivers in the BBC program. Any thoughts about why this is happening?
   - What are some challenges to enrollment?
   - What could be done to increase or improve the enrollment process?
   - What staff members at your VA work with Veterans with caregivers? (e.g., SCI, TBI, OT, extended care)
2. Do you have any suggestions about how to include more caregivers of pre-9/11 Vets?

**Resources and Leadership Support of BBC**

1. What are your thoughts about how staff and resources are being used for the BBC program?
   - 1. What additional resources are needed?
2. What are your facility’s leadership attitudes about the program? (supportive, unsupportive)
   1. Who is your leadership?

**Staff Attitudes about BBC**

1. How do other staff at your VA feel about the BBC program?

**Caregiver and Veteran Attitudes about BBC**

1. Based on your experience with the program, what impact has it had?
   - 1. On caregivers?
     2. On Veterans?
     3. On staff?
2. Have you asked caregivers for feedback about the program?
   1. If yes, What did they say?
      1. Challenges related to the program? (e.g., enrollment, participation)
      2. How could these challenges be resolved?
      3. Benefits of the program?
      4. Limitations of the program?
   2. If no, what kind of feedback would be helpful?
3. How could the BBC program be more appealing to caregivers?

**Conclusion**

*Thanks so much for talking with me today.*

*We really appreciate you taking the time to share your experiences and input.*

1. Anything else we should know about your experiences with the BBC program?
2. Anything we forgot to ask you about or that you would like to add to our discussion?

**REGIONAL & NATIONAL LEADERSHIP *-* SEMISTRUCTURED INTERVIEW GUIDE**

The focus of this interview is to understand leadership perceptions of and experiences with the Building Better Caregivers program in the VHA. By talking with you, we hope to learn about how the program is working so we can help evaluate what is working well and what might not be working well. One overall goal is to contribute to the process of implementing the program to boost enrollment and to improve caregivers’ experience with it.

Just a few reminders before we get started. Feel free to ask questions as we go along. There are no right or wrong answers. If you feel uncomfortable with any question or need to take a break, let me know. What we talk about today will be kept confidential and private. We'd like to audio record the interview to make sure we keep good track of our discussion today. Do you have questions before we get started?

Is it ok with you if I turn on the audio recorder?

- Yes
- No

*[If yes, begin recording. Be prepared to take notes should the participant refuse audio recording.]*

**Roles and Perspectives related to Caregiver Support Program and BBC**

*I’d like to learn more about you and your experience with the Caregiver Support Program.*

1. What is your involvement with the Caregiver Support Program?

**Belief about Evidence for Use of BBC**

*For the next part of our discussion, I would like to learn about your experience with the Building Better Caregivers (or “BBC”) program. First, I would like understand whether you believe the BBC program is based on good research.*

1. Do you think there is good evidence that the BBC program helps caregivers? Why or why not?

**BBC Role**

*The BBC program first became available to caregivers of Veterans in January 2013.*

1. How involved were you in the initial rollout of the program in 2013?
   1. Can you tell me what you contributed to that rollout?
2. Tell me about your recent experience with the BBC program.
   1. What is your role in the program and referrals to it?
   2. How does your role with the BBC program fit/not fit with your other work responsibilities?
   3. What do you think about your level of involvement with the program? (enough, too much, too little)

**BBC Processes**

*From what you have described, it sounds like you have been involved in BBC processes at the [national, regional, and/or local level(s)] of the VHA. [Allow time for confirmation]*

1. I would like to learn more about how BBC processes work at [that/those] levels of operation.
   1. What is the process for informing VA staff members about the availability of the BBC program for caregivers?
   2. What is the process of informing caregivers about the program?
   3. What is the process of referring caregivers to the program
      1. Are there ways in which these processes can or should be streamlined?
2. Is there an expectation that BBC program receive regular referrals? If yes, what exactly is the expectation?

**Enrollment Challenges**

*I would now like to shift our conversation a little to talk about your perspective on the BBC program implementation.*

1. From your perspective, how is the implementation of the program going?
   1. What have been the easiest things to accomplish?
   2. What have been some of the challenges to having the program reach the right caregivers?
   3. What lessons have you learned?
   4. Have there been any changes to the implementation process? If so, what are the changes?
2. Some VA sites have had more challenges enrolling caregivers in the BBC program while others have had less.
   1. Why do you think that is?
   2. What are some of the challenges to enrollment?
   3. What do you think could be done to increase enrollment in the BBC program?

**Leadership Support and Facilitators**

1. What do you think about the current allocation of resources related to the BBC program?
   1. Are any additional resources needed?
      1. Probe: other people that need to be involved, money, time, IT support
   2. What kinds of additional resources are needed as the program is scaled up?
2. Who do you consider to be the leaders of the BBC program?
   1. How do you interact with them?
   2. Is there anyone who helps you when you interact with the leadership?
      1. Who? What is his/her process for doing this?
      2. Has it been been helpful?
3. How has (local, regional, national) leadership supported the BBC program?
4. What happens if the program is unsuccessful? Who is held accountable?
5. Do you feel personally responsible for the success of the program? Why is that?
6. What kinds of things would help maintain the BBC program over time?

**Evaluation**

*I want to understand how the BBC program is being evaluated. Examples might be the number of referrals made, caregivers who have completed the program, caregiver feedback on program content, or other things.*

1. How is the BBC program currently being evaluated?
   1. Is there other information that would be useful to have to shed light on what is going on with the program?
   2. What is the best way to collect this information?
   3. Who should be responsible for collecting this information?
   4. What about the on-the-ground staff—what feedback have they given that has been useful for evaluating the BBC program implementation?

*I now want to learn about what is at stake with these evaluations of the BBC.*

1. What kinds of decisions get made based on the evaluations?
   1. Probe: national, regional, local levels

**Clinical Experience**

1. We are trying to get a sense of how staff members feel in general about the BBC program. What do other staff members think of the BBC program?

**Participant Experience**

1. Based on your understanding of the program, what impact has it had?
   1. On caregivers?
   2. On Veterans?
   3. On staff?
2. Have you had the opportunity to talk with caregivers about the program? If yes,
   1. What have been caregivers’ reactions to the program?
   2. Challenges related to the program? (e.g., enrollment, participation)
   3. How could these challenges be resolved?
   4. Benefits of the program?
   5. Limitations of the program?

**Conclusion**

1. Is there anything else we should know about your experiences with the BBC program?
2. Anything we forgot to ask you about or that you would like to add to our discussion?

*Thanks so much for talking with me today.*

*We really appreciate you taking the time to share your experiences and input.*
