# Supplementary material for: Improving Uptake of a National Web-Based Psychoeducational Workshop for Informal Caregivers of Veterans: Mixed Methods Implementation Evaluation
Source: J Med Internet Res. 2021 Jan 7;23(1):e16495. doi: 10.2196/16495 (PMC7819783; doi:10.2196/16495)
Supplement: Multimedia Appendix 2 [file jmir_v23i1e16495_app2.pdf]

Building Better Caregivers Participant Survey

Thank you for agreeing to participate in our pilot evaluation of VA's roll out of the Building Better Caregivers (BBC) program. By roll out, we mean the process of educating, recruiting, enrolling, and assessing caregiver participation in the program. The purpose of this survey is to help us understand a bit about you as a caregiver and how others like you may benefit from the BBC program. We will use this information to look for patterns across VA facilities that may be associated with better or worse program enrollment. We also will use the information to design a future large-scale study and one or more interventions to boost caregiver enrollment in the program.

For this part of the evaluation, you will complete survey questions that will help us gather some basic background information about your role as a caregiver. This part should take 15 to 30 minutes to complete. It is voluntary, and your input will be treated as confidential. To ensure confidentiality, a unique ID number will be associated with your information rather than your name.

CONTINUE TO THE NEXT PAGE

I. About the Veteran you care for

1. How old is the Veteran you care for? ____________years old

2. Is the Veteran you care for? (check **one**)
   - ☐ Male
   - ☐ Female

3. Is the Veteran you care for? (check **all** that apply)
   - ☐ White/Caucasian
   - ☐ Black or African-American
   - ☐ American Indian or Alaskan Native
   - ☐ Asian
   - ☐ Native Hawaiian or other Pacific Islander
   - ☐ Other, please specify: ____

4. What is the ethnicity of the Veteran you care for? (check **one**)
   - ☐ Hispanic
   - ☐ Not Hispanic

5. Within the past 12 months, through what provider(s) has the Veteran received health care? (check **one**)
   - ☐ The VA
   - ☐ A non-VA health provider
   - ☐ Both
   - ☐ Neither – The Veteran has not had health care in the past 12 months
   - ☐ Don't know

6. What is the Veteran's relationship to you? Is he or she your… (check **one**)
   - ☐ Spouse
   - ☐ Partner or significant other
   - ☐ Son or daughter
   - ☐ Son-in-law or daughter-in-law
   - ☐ Parent (father or mother)
   - ☐ Parent-in-law (father-in-law or mother-in-law)
   - ☐ Sibling (brother or sister)
   - ☐ Sibling-in-law (brother-in-law or sister-in-law)
   - ☐ Grandparent or great grandparent
   - ☐ Uncle or Aunt, or great uncle/aunt
   - ☐ Friend/neighbor
   - ☐ Other relative
   - ☐ Other non-relative

CONTINUE TO THE NEXT PAGE

7. When did you first take on a caregiving role? (check **one**)

   - ☐ Within the first few months that the Veteran began needing care
   - ☐ Some time later

8. How long have you been providing care for the Veteran? (check **one**)
   - ☐ Less than one year
   - ☐ 1 to 3 years
   - ☐ 4 to 10 years
   - ☐ More than 10 years

9. Are you paid to provide care for the Veteran? (check **one**)
   - ☐ Yes
   - ☐ No
   - ☐ Maybe/Not sure

CONTINUE TO THE NEXT PAGE

10. Does the Veteran you care for have: (check **all** that apply)

- ☐ Post-traumatic stress disorder (PTSD)
- ☐ Mental illness or illnesses — please check all that apply, if known:
  - ☐ Depression
  - ☐ Anxiety
  - ☐ Schizophrenia or schizoaffective disorder
  - ☐ Bipolar disorder
  - ☐ Obsessive compulsive disorder
  - ☐ Other (please describe):____
  - ☐ Don't know/not sure
- ☐ Addiction to alcohol or other drugs— please check all that apply, if known:
  - ☐ Alcohol
  - ☐ Prescription drugs (e.g., painkillers)
  - ☐ Other drugs (e.g., cocaine, methamphetamine, marijuana)
  - ☐ Don't know/not sure
- ☐ Neurological condition – please check all that apply, if known:
  - ☐ Stroke
  - ☐ Traumatic brain injury (TBI)
  - ☐ Paralysis or spinal cord injury (SCI)
  - ☐ Multiple sclerosis (MS)
  - ☐ Alzheimer's disease or another form of dementia
  - ☐ Parkinson's disease
  - ☐ Don't know/not sure
- ☐ Other chronic medical condition – please check all that apply, if known:
  - ☐ Any amputated hands, arms, feet or legs
  - ☐ Cancer (e.g., lung cancer, leukemia, melanoma, etc.)
  - ☐ Blindness
  - ☐ Diabetes
  - ☐ Hypertension
  - ☐ Heart failure
  - ☐ Other heart disease (e. g., coronary artery disease)
  - ☐ Kidney failure/disease
  - ☐ Liver failure/disease
  - ☐ Asthma or chronic obstructive pulmonary disease (COPD)
  - ☐ Serious joint impairment (e.g., severe arthritis)
  - ☐ Chronic pain
  - ☐ Other conditions, please specify: ____
  - ☐ Don't know/not sure

CONTINUE TO THE NEXT PAGE

11. As far as you know, has the Veteran you care for ever been exposed to any of the following types of events? (check **all** that apply)
    - ☐ Combat
    - ☐ Sexual assault or severe sexual harassment
    - ☐ Violence or assault (not including combat)
    - ☐ Life-threatening accident or illness
    - ☐ Childhood abuse or neglect

II. Your personal health, well-being, and experiences

12. In general, would you say your health is....(check **one**)
    - ☐ Excellent
    - ☐ Very good
    - ☐ Good
    - ☐ Fair
    - ☐ Poor

13. How emotionally stressful would you say that caring for the Veteran is for you? (check **one**)
    - ☐ Not at all stressful
    - ☐ Slightly stressful
    - ☐ Somewhat stressful
    - ☐ Very stressful
    - ☐ Extremely stressful

14. How much of a physical strain would you say that caring for the Veteran is for you? (check **one**)
    - ☐ Not at a strain at all
    - ☐ A slight strain
    - ☐ Somewhat of a strain
    - ☐ Very much of a strain
    - ☐ An extreme strain

CONTINUE TO THE NEXT PAGE

15. As a result of becoming a caregiver, which of the following health problems, if any, have you experienced? (check **all** that apply)
    - ☐ Increased blood pressure
    - ☐ Weight gain or loss
    - ☐ Increased stress or anxiety
    - ☐ Sleep deprivation
    - ☐ Misuse of alcohol or drugs
    - ☐ Depression
    - ☐ Generally getting sick more often
    - ☐ Poor eating habits
    - ☐ Delaying or skipping your own doctor/dentist appointments
    - ☐ Less time exercising
    - ☐ Strains, aches, pains
    - ☐ Other health problems (Please specify: ______________________________________)
    - ☐ None of the above

16. As a result of caring for your Veteran, how much you agree or disagree with each of the following statements? (check **one** for each question)

	Disagree a lot	Disagree a little	Neither agree nor disagree	Agree a little	Agree a lot
a. Caregiving has made me feel more useful					
b. Caregiving has made me feel good about myself					
c. Caregiving has made me feel more needed					
d. Caregiving has made me feel more appreciated					
e. Caregiving has made me feel more important					
f. Caregiving has made me feel more strong and confident					
g. Caregiving has enabled me to appreciate life more					
h. Caregiving has enabled me to develop a more positive attitude toward life					
i. Caregiving has strengthened my relationships with others					

CONTINUE TO THE NEXT PAGE

17. Over the past 2 weeks, how often have you been bothered by any of the following problems? (check **one** for each question)

	Not at all	Several days	More than half the days	Nearly every day
a. Little interest or pleasure in doing things?				
b. Feeling down, depressed, or hopeless?				
c. Trouble falling or staying asleep, or sleeping too much?				
d. Feeling tired or having little energy?				
e. Poor appetite or over eating?				
f. Feeling bad about yourself or that you are a failure, or have let yourself or your family down?				
g. Trouble concentrating on things, such as reading the newspaper watching television?				
h. Moving or speaking so slowly that other people could have noticed. Or the opposite – being so fidgety or restless that you have been moving around a lot more than usual?				
i. How often during the past 2 weeks were you bothered by thoughts that you would be better off dead, or of hurting yourself in some way?				

CONTINUE TO THE NEXT PAGE

III. Caregiving skills and activities

18. With which of the following tasks do you help the Veteran? (check **all** that apply)
    - ☐ Getting in and out of beds and chairs
    - ☐ Getting dressed
    - ☐ Getting to and from the toilet
    - ☐ Bathing or showering
    - ☐ Dealing with incontinence or diapers
    - ☐ Feeding him or her
    - ☐ Giving medicines, pills, or injections
    - ☐ Managing finances (such as bills or insurance paperwork)
    - ☐ Grocery shopping
    - ☐ Housework (such as dishes, laundry, or straightening up)
    - ☐ Preparing meals
    - ☐ Transportation (driving, helping arrange for transportation, or accompanying on public transit)
    - ☐ Arranging or supervising paid services (such as nurses, aides, Meals on Wheels, or other services)
    - ☐ Coordinating medical care and rehabilitation services
    - ☐ Advocating for him with care providers, government agencies, or schools
    - ☐ Administering physical or medical therapies or treatments
    - ☐ Reminding or giving cues about what he he/she should be doing or helping with memory tasks
    - ☐ Helping him or her cope with stressful situations or avoid “triggers” of anxiety or anti-social behavior
    - ☐ Working with him or her to minimize disruptive behaviors

19. In a typical week, about how many hours in *total* do you spend helping the Veteran in *all* of the ways you just indicated? (Your best estimate is fine.) (check **one**)
    - ☐ Less than one hour
    - ☐ 1 to 4 hours
    - ☐ 5 to 8 hours
    - ☐ 9 to 20 hours
    - ☐ 21 to 30 hours
    - ☐ 31 to 40 hours
    - ☐ 41 to 60 hours
    - ☐ 61 to 80 hours
    - ☐ More than 80 hours

CONTINUE TO THE NEXT PAGE

20. How much caregiving has been provided to the Veteran during the past 12 months by…? (check **one** for each question)

	None	A little	A moderate amount	A great deal	Don't know
a. **Paid** family members and friends other than you					
b. **Unpaid** family members and friends other than you					
c. Other **paid caregivers** who help with daily activities (e.g., home health aide/nurse, personal assistant, adult day care, respite providers)					
d. Other **unpaid caregivers** who help with daily activities (e.g., volunteers from service organizations)					

21. In the past 12 months, have <u>you</u> received any respite services (short-term or temporary relief from caregiving) from the VA or any other organizations? (check **one**)
- ☐ Yes
- ☐ No
- ☐ Maybe/Not sure

22. How confident in your caregiving skills did you feel at these points in time? (check **one** for each question)

	Not at all confident	A little confident	Somewhat confident	Very confident
a. During your first six months as a caregiver				
b. Nowadays				

CONTINUE TO THE NEXT PAGE

23. Have you looked for the following resources that may be available to you or the Veteran from any community or governmental organizations? If yes, how easy or difficult were they to find… (check **one** for each question)

	Have you looked?			How easy or difficult to find?			
	Yes	No		Very difficult	Somewhat difficult	Somewhat easy	Very easy
a. Resources/services you need for the Veteran to coordinate his or her care							
b. Resources/services for your own health and well-being							
c. Resources/services to give you caregiver-related training or education							

CONTINUE TO THE NEXT PAGE

24. Did you turn to any of the following avenues to look for caregiver resources and information? If yes, how helpful was this source? (check **one** for each question)

	Did you turn to…?		How helpful was it?			
	Yes	No	Not at all helpful	Not too helpful	Somewhat helpful	Very helpful
a. Word-of-mouth or asking around						
b. Support groups (in person)						
c. Blogs, online forums, or online groups						
d. The VA Benefits Administration						
e. The VA Health Administration						
f. Disease-specific organizations (e.g., M.S. Society, Brain Injury Foundation, American Diabetes Association, American Cancer Society, etc.)						
g. A non-VA health provider (e.g., a private doctor, clinic, hospital, or insurance company)						
h. Military OneSource						
i. Local government or community organizations (e.g., aging organizations, county programs, churches, Rotary Club, etc.)						
j. National Resource Directory website						
k. The D.O.D. military system						

CONTINUE TO THE NEXT PAGE

25. Have you personally experienced the following challenges at any point since you became a caregiver? If yes, to what degree? (check **one** for each question)

	Have you experienced this challenge?			
	No, not yet	Yes, to a minor degree	Yes, to a major degree	Not applicable
a. Not knowing what to expect medically with the Veteran's illness or condition				
b. Not knowing how to properly administer medical treatments, care, or medications				
c. Not knowing how to address certain behaviors or problems related to his or her PTSD, mental illness or other disruptive behaviors				
d. Not being aware of services VA has that can help you or your Veteran				
e. Not knowing where to obtain specialized care for Veterans such as rehabilitation, occupational therapy, speech therapy, etc.				
f. Not knowing how to arrange or pay for home modifications (e.g., ramps, grab bars, widened doors)				
g. Not knowing where to turn in order to arrange a break from caregiving from time to time				
h. Not knowing where to turn to obtain financial assistance				
i. Not knowing best how to plan the Veteran's transition into or out of different care facilities or locations				
j. Difficulty getting through bureaucracy in order to obtain services from the VA				
k. Being excluded from being a key player in decisions about the Veteran's care				

CONTINUE TO THE NEXT PAGE

IV. Impact of caregiving

26. Please indicate how much you agree or disagree with each statement. (check **one** for each question)

	Disagree strongly	Disagree somewhat	Agree somewhat	Agree strongly
a. You are proud of the support and assistance you provide				
b. Gaining new knowledge and skills from caregiving feels rewarding				
c. The experience of caregiving has been fulfilling for you				
d. Caring for your Veteran has altered your ideas about what is important in life				
e. You have developed a closer relationship with your Veteran since you have been caring for him/her				
f. You feel isolated				
g. You do not have enough time for yourself				
h. You feel you don't have a life of your own anymore				
i. You hesitate to take your Veteran anywhere because you are afraid of what might happen				
j. You feel no one else really understands what you're going through				
k. You feel you are the only person who can provide adequate care to the Veteran				

27. Do you feel you had a choice in taking on the responsibility of caring for the Veteran? (check **one**)
    - ☐ Yes
    - ☐ No
    - ☐ Don't know

CONTINUE TO THE NEXT PAGE

28. How often do you feel… (check **one** for each question)

	Never	Rarely	Sometimes	Quite frequently	Nearly always
a. That because of the time you spend with your Veteran that you don't have enough time for yourself?					
b. Stressed between caring for your Veteran and trying to meet other responsibilities (work/family)?					
c. Angry when you are around your Veteran?					
d. That your Veteran currently affects your relationship with family members or friends in a negative way?					
e. Strained when you are around your Veteran?					
f. That your health has suffered because of your involvement with your Veteran?					
g. That you don't have as much privacy as you would like because of your Veteran?					
h. That your social life has suffered because you are caring for your Veteran?					
i. That you plan your life around your Veteran?					
j. Uncertain about what to do about your Veteran?					
k. You should be doing more for your Veteran?					
l. You could do a better job in caring for your Veteran?					

CONTINUE TO THE NEXT PAGE

29. As a result of the Veteran's condition or your caregiving, has the following occurred? (check **one** for each question)

	Yes	No	Not applicable
a. You had to move to a new city/town			
b. You have spent less time with your children/grandchildren than you would like to			
c. Your children or grandchildren experienced emotional problems or school problems			
d. You spent less time with friends and family			
e. Strain was placed on your relationship with a spouse or significant other			

30. If you ever felt you needed to take a break (respite) from your caregiving, how easy or difficult would it be for you to get someone else to take on your caregiving responsibilities? (check **one**)
    - ☐ Very difficult
    - ☐ Somewhat difficult
    - ☐ Somewhat easy
    - ☐ Very easy
    - ☐ Haven't needed to take a break from caregiving
    - ☐ Not applicable, Veteran can be alone without a replacement

31. Other than caring for the Veteran, are you currently employed full-time, part-time, or are you not currently employed? (check **one**)
    - ☐ Full-time
    - ☐ Part-time
    - ☐ Not currently employed

32. Since you began caregiving, have you **ever** had other paid employment? (check **one**)
    - ☐ Yes
    - ☐ No

CONTINUE TO THE NEXT PAGE

33. How much of a financial hardship would you say that caring for the Veteran is for you? (check **one**)
    - ☐ No hardship at all ➜
        - ☐ My financial situation *has improved* since I began caring for the Veteran
        - ☐ My financial situation *has not changed* since I began caring for the Veteran
    - ☐ A slight hardship
    - ☐ Somewhat of a hardship
    - ☐ Very much of a hardship
    - ☐ An extreme hardship

34. As a result of caregiving for the Veteran, did you ever… (check **one** for each question)

	Yes	No	Not applicable
a. Take unpaid time off from work or stop working temporarily			
b. Cut back the number of hours in your regular weekly job schedule			
c. Move to a job that pays less or provides fewer benefits, but that fits better with your caregiving schedule or responsibilities			
d. Quit working entirely or take retirement earlier than you would have otherwise			
e. Give up or postpone plans that you had to further your own education			
f. Reduce or stop saving for your own future			
g. Reduce or stop saving for your children's future			
h. Delay a major purchase such as buying a care, furniture, a home improvement, or some other major purchase			

CONTINUE TO THE NEXT PAGE

35. Did you complete the Building Better Caregivers Program (BBC)? (check **one**)

- ☐ No (skip to question 36)
- ☐ Yes

  Have you saved time as a result of what you learned from the program?
  - ☐ No
  - ☐ Yes
  - ☐ Maybe/not sure

  Have you saved money as a result of what you learned from the program?
  - ☐ No
  - ☐ Yes
  - ☐ Maybe/not sure

  Has your quality of life improved as a result of what you learned from the program?
  - ☐ No
  - ☐ Yes
  - ☐ Maybe/not sure

  Would you recommend the program to other caregivers?
  - ☐ No
  - ☐ Yes
  - ☐ Maybe/not sure

36. Other than the Building Better Caregivers Program (BBC), have you obtained any formal training about how to care for someone with your Veteran's needs? (check **one**)

- ☐ Yes, please specify: ____
- ☐ No
- ☐ Maybe/Not sure

V. About You

37. What is your Marital Status? (check **one**)

- ☐ Single
- ☐ Married
- ☐ Significant other
- ☐ Divorced
- ☐ Separated
- ☐ Widowed

CONTINUE TO THE NEXT PAGE

38. Do you live with your Veteran? (check **one**)
- ☐ Yes
- ☐ No

39. Are you male or female? (check **one**)
- ☐ Male
- ☐ Female

40. What is your Race? (check **all** that apply)
- ☐ White/Caucasian
- ☐ Black or African-American
- ☐ American Indian or Alaskan Native
- ☐ Asian
- ☐ Native Hawaiian or other Pacific Islander
- ☐ Other, please specify: ____

41. What is your ethnicity? (check **one**)
- ☐ Hispanic
- ☐ Not Hispanic

42. How old are you? (check **one**)
- ☐ 17 or younger
- ☐ 18-20 years old
- ☐ 21-29 years old
- ☐ 30-39 years old
- ☐ 40-49 years old
- ☐ 50-59 years old
- ☐ 60 or older

Thank you!

Please return your completed survey using the enclosed postage-paid envelope.
