# Supplementary material for: Improving Uptake of a National Web-Based Psychoeducational Workshop for Informal Caregivers of Veterans: Mixed Methods Implementation Evaluation
Source: J Med Internet Res. 2021 Jan 7;23(1):e16495. doi: 10.2196/16495 (PMC7819783; doi:10.2196/16495)
Supplement: Multimedia Appendix 3 [file jmir_v23i1e16495_app3.pdf]

Part. ID: _________ Date: ___/___/___

Building Better Caregivers (BBC) Staff Survey

Thank you for agreeing to participate in our pilot evaluation of VA's roll out of the Building Better Caregivers (BBC) program. By roll out, we mean the process of educating, recruiting, enrolling, and monitoring caregiver participation in the program. The purpose of this survey is to help us understand some of the local factors that may contribute to observed differences in how the program has been rolled out across the VA. Information from this pilot evaluation will be used by the research team to design a future large-scale study and design one or more interventions to boost enrollment in the BBC program.

This survey is designed to be completed in ~15 minutes. It is voluntary, and your input will be treated as confidential. Results of the survey will be used solely for evaluation and planning purposes. To ensure confidentiality, reports and publications will be provided only as grouped responses.

If you have any questions or comments about this survey, please contact Randy Gale, Program Analyst, at Randall.Gale@va.gov or 650-493-5000 (the press "1" followed by "2" followed by extension "27325").

You must complete the survey in one session. If you exit the survey early, your place in the survey will not be saved and you will be required to start the survey from the beginning.

In order to progress through this survey, please use the following navigation links:

- Click the "Next" button to continue to the next page.
- Click the "Prev" button to return to the previous page.
- On the last page of the survey, click the "Submit" button to submit the survey.

Click on the "Next" button to begin the survey.

Part. ID: _________ Date: ___/___/___

I. Caregiver Support Services at Your Facility

For each of the following statements please rate (circle) the strength of your agreement with the statement, from 1 (strongly disagree) to 5 (strongly agree).

	**In general at my facility, when there is agreement on implementing new caregiver support services:**	strongly disagree	disagree	neither agree nor disagree	agree	strongly agree	Don't know/ Not applicable
1	we have the necessary support in terms of budget or financial resources	1	2	3	4	5	99
2	we have the necessary support in terms of training	1	2	3	4	5	99
3	we have the necessary support in terms of facilities	1	2	3	4	5	99
4	we have the necessary support in terms of staffing	1	2	3	4	5	99

CONTINUE TO THE NEXT PAGE

**Senior leadership/management in your facility:**	strongly disagree	disagree	neither agree nor disagree	agree	strongly agree	Don’t know/ Not applicable
5 provides staff with information on VA performance measures and guidelines related to caregiver support services	1	2	3	4	5	99
6 establishes clear goals for caregiver support services	1	2	3	4	5	99
7 provides staff members with feedback/data on the effects of caregiver support services	1	2	3	4	5	99
8 holds staff members accountable for achieving results related to caregiver support services	1	2	3	4	5	99

**Senior leadership/management in your facility:**	strongly disagree	disagree	neither agree nor disagree	agree	strongly agree	Don’t know/ Not applicable
9 rewards innovation and creativity for improving caregiver support services	1	2	3	4	5	99
10 solicits staff opinions regarding decisions about caregiver support services	1	2	3	4	5	99
11 seeks ways to improve caregiver education	1	2	3	4	5	99
12 seeks ways to increase caregiver engagement in care of Veterans	1	2	3	4	5	99
13 provides effective management for continuous improvement of caregiver support services	1	2	3	4	5	99

CONTINUE TO THE NEXT PAGE

	**Opinion leaders in your facility:**	strongly disagree	disagree	neither agree nor disagree	agree	strongly agree	Don't know/ Not applicable
14	believe current caregiver support services can be improved	1	2	3	4	5	99
15	encourage and support changes to improve caregiver support services	1	2	3	4	5	99
16	work cooperatively with senior leadership/management to make appropriate changes to caregiver support services	1	2	3	4	5	99

	**Staff members in your facility:**	strongly disagree	disagree	neither agree nor disagree	agree	strongly agree	Don't know/ Not applicable
17	have a sense of personal responsibility for improving caregiver engagement	1	2	3	4	5	99
18	have a sense of personal responsibility for improving caregiver health and well-being	1	2	3	4	5	99
19	cooperate to maintain and improve effectiveness of caregiver support services	1	2	3	4	5	99
20	are willing to innovate and/or experiment to improve caregiver support services	1	2	3	4	5	99
21	refer patients and their caregivers to the caregiver support services	1	2	3	4	5	99

CONTINUE TO THE NEXT PAGE

Part. ID: _________ Date: ___/___/___

II. Building Better Caregivers Program Implementation at Your Facility

For each of the following statements please rate (circle) the strength of your agreement with the statement, from 1 (strongly disagree) to 5 (strongly agree).

	**The BBC program:**	strongly disagree	disagree	neither agree nor disagree	agree	strongly agree	Don’t know/ Not applicable
22	is supported by research/evidence	1	2	3	4	5	99
23	should be effective, based on current scientific knowledge	1	2	3	4	5	99
	**…for caregivers,**						
24	takes into consideration the needs and preferences of VA caregivers	1	2	3	4	5	99
25	has been well-accepted by VA caregivers	1	2	3	4	5	99
26	has improved outcomes for caregivers of Veterans with serious chronic conditions	1	2	3	4	5	99
27	appears to have more advantages than disadvantages for VA caregivers	1	2	3	4	5	99
	**…for VA staff,**						
28	takes into consideration the needs and preferences of VA staff	1	2	3	4	5	99
29	has been well-accepted by VA staff	1	2	3	4	5	99

	**Senior leadership/management/staff opinion leaders:**	strongly disagree	disagree	neither agree nor disagree	agree	strongly agree	Don’t know/ Not applicable
30	understand the goals of the program	1	2	3	4	5	99
31	have been involved in program implementation	1	2	3	4	5	99
32	provide adequate resources for implementation	1	2	3	4	5	99
33	set a high priority on the success of the program	1	2	3	4	5	99
34	have realistic expectations for program implementation	1	2	3	4	5	99

CONTINUE TO THE NEXT PAGE

	At our facility:	strongly disagree	disagree	neither agree nor disagree	agree	strongly agree	Don't know/ Not applicable
35	there is a BBC program champion (i.e., someone who has taken extraordinary interest in the program's success)	1	2	3	4	5	99
36	I am the local BBC program champion	1	2	3	4	5	99

	Our local facility's BBC program champion:	strongly disagree	disagree	neither agree nor disagree	agree	strongly agree	Don't know/ Not applicable
37	accepts responsibility for program success	1	2	3	4	5	99
38	has the authority to carry out program implementation	1	2	3	4	5	99
39	is considered a facility opinion leader	1	2	3	4	5	99
40	works well with the implementation team	1	2	3	4	5	99

	The BBC implementation team members:	strongly disagree	disagree	neither agree nor disagree	agree	strongly agree	Don't know/ Not applicable
41	share responsibility for the success of the program	1	2	3	4	5	99
42	have clearly defined roles and responsibilities	1	2	3	4	5	99
43	have protected time or can accomplish implementation tasks within their regular work load	1	2	3	4	5	99
44	have adequate staff support and other resources needed for implementation	1	2	3	4	5	99

CONTINUE TO THE NEXT PAGE

	As a member of the BBC implementation team:	strongly disagree	disagree	neither agree nor disagree	agree	strongly agree	Don't know/ Not applicable
45	I am responsible for educating caregivers about the program	1	2	3	4	5	99
46	I am responsible for referring caregivers to the program	1	2	3	4	5	99
47	I am responsible for educating other VA staff members/providers about the program	1	2	3	4	5	99
48	I am responsible for monitoring success of the program's rollout	1	2	3	4	5	99
49	my contributions are valued by the implementation team	1	2	3	4	5	99
50	I am responsible for program success	1	2	3	4	5	99

	Communication about implementation of the program has been maintained through:	strongly disagree	disagree	neither agree nor disagree	agree	strongly agree	Don't know/ Not applicable
51	regular meetings with the program champion and implementation team members	1	2	3	4	5	99
52	involvement of quality management/improvement team in program planning and implementation	1	2	3	4	5	99
53	regular feedback to senior leadership/management on implementation progress and resource needs	1	2	3	4	5	99

CONTINUE TO THE NEXT PAGE

	The following resources are available to make BBC program implementation work:	strongly disagree	disagree	neither agree nor disagree	agree	strongly agree	Don't know/ Not applicable
	**Materials**						
54	for implementation team members to support program roll-out	1	2	3	4	5	99
55	for implementation team members about the general structure and content of the program	1	2	3	4	5	99
56	to educate implementation team members about how to make referrals to the program	1	2	3	4	5	99
57	informational materials for other VA staff to raise awareness about the program	1	2	3	4	5	99
58	informational materials for caregivers to raise awareness about the program	1	2	3	4	5	99
	**People**						
59	A contact person outside of the VA who can answer questions or help solve problems related to the program	1	2	3	4	5	99
60	A regional VA representative who can answer questions or help solve problems related to the program	1	2	3	4	5	99
61	A national VA representative who can answer questions or help solve problems related to the program	1	2	3	4	5	99

CONTINUE TO THE NEXT PAGE

	Approaches used at our facility to evaluate and improve BBC program implementation have included:	strongly disagree	disagree	neither agree nor disagree	agree	strongly agree	Don't know/ Not applicable
60	system of tracking which caregivers have been referred to the program	1	2	3	4	5	99
61	system of tracking which caregivers have enrolled in the program	1	2	3	4	5	99
62	review of referral or enrollment results by the implementation team	1	2	3	4	5	99
63	review of referral or enrollment results by senior leadership/management	1	2	3	4	5	99
64	staff satisfaction surveys	1	2	3	4	5	99
65	caregiver satisfaction surveys	1	2	3	4	5	99
66	staff interviews (formal or informal)	1	2	3	4	5	99
67	caregiver interviews (formal or informal)	1	2	3	4	5	99
68	review of survey or interview results by the implementation team	1	2	3	4	5	99
69	review of survey or interview results by senior leadership/management	1	2	3	4	5	99

	At our facility, implementation of the BBC program:	strongly disagree	disagree	neither agree nor disagree	agree	strongly agree	Don't know/ Not applicable
70	has been a success	1	2	3	4	5	99

III. About You

71. What is your gender?
    a. Male
    b. Female

72. How old are you? ________

Thank you!

Please return your completed survey to a member of the evaluation team.
