# Supplementary material for: Improving Uptake of a National Web-Based Psychoeducational Workshop for Informal Caregivers of Veterans: Mixed Methods Implementation Evaluation
Source: J Med Internet Res. 2021 Jan 7;23(1):e16495. doi: 10.2196/16495 (PMC7819783; doi:10.2196/16495)
Supplement: Multimedia Appendix 4 [file jmir_v23i1e16495_app4.docx]

**Appendix 4. Available VA operations data on characteristics of caregivers enrolled in BBC workshop during rollout years 1-2 and their veteran care partners**

Operations data from years 1-2 of the Building Better Caregivers (BBC) workshop rollout are shown alongside data from the current implementation evaluation for ease of comparison.

| **Table A. Caregiver relationship with veteran care partner** | | |
| --- | --- | --- |
| **Relationship** | **Caregivers enrolled in BBC^a^ during rollout years 1-2**  (n=1607) | **Caregivers in current implementation evaluation**  (n=32) |
| Spouse/significant other | 86% | 81% |
| Child | 8% | 9% |
| Sibling | 1% | 6% |
| Parent | 5% | 3% |
| Other relationship | 1% | 0 |
| ^a^BBC: Building Better Caregivers. | | |

| **Table B. Health conditions of veteran care partner as reported by caregivers** | | | |
| --- | --- | --- | --- |
|  |  | **Care partners of caregivers enrolled in BBC during rollout years 1-2**  (n=1607) | **Care partners of caregivers in current implementation evaluation**  (n=32) |
| Most common chronic conditions^a^ | PTSD^b^  Other mental health^c^  -Depression  -Anxiety  TBI^d^  Dementia  Other medical^c^  -Chronic pain  -Hypertension  -Joint disease  -Diabetes  -COPD^e^ | 78%  49%^b^    48%  10%  22%^b^ | 74%  82%  82%  65%  47%  21%  94%  77%  53%  29%  18%  18% |
| Multi-morbidity | 2+ conditions  3+ conditions | Not available | 100%  95% |
| ^a^Conditions listed here are those present in 15% or more of veteran partners of the caregivers who responded to the implementation evaluation survey.  ^b^PTSD: post-traumatic stress disorder.  ^c^The Caregiver Support Program operations survey of caregivers enrolled in the Building Better Caregivers workshop during rollout years 1-2 contained a much more limited list of health conditions to select from than our implementation evaluation survey of caregivers, which may explain the observed differences in reported rates of “other mental health” conditions and “other medical” conditions.  ^d^TBI: traumatic brain injury.  ^e^COPD: chronic obstructive pulmonary disease. | | | |
