# Supplementary material for: Improving Uptake of a National Web-Based Psychoeducational Workshop for Informal Caregivers of Veterans: Mixed Methods Implementation Evaluation
Source: J Med Internet Res. 2021 Jan 7;23(1):e16495. doi: 10.2196/16495 (PMC7819783; doi:10.2196/16495)
Supplement: Multimedia Appendix 5 [file jmir_v23i1e16495_app5.docx]

**Appendix 5. Detailed description of implementation changes made by VA in response to stage 1 implementation evaluation findings and recommendations**

This table is organized according to the qualitative themes listed in manuscript Table 4, so that the information below can be aligned with Table 4 results. Data here can also be contextualized by data from manuscript Table 3 on survey results because the last column of Table 3 indicates whether survey findings are consistent with the qualitative themes from interviews.

| **Table. VA^a^ actions taken in response to initial implementation evaluation findings** | |
| --- | --- |
| **Finding** | **Actions** |
| 1. Importance of outreach and marketing | Organization that administers workshop changed to one with robust outreach/marketing skill set |
| 2. Belief in positive impact of workshop | Peer beliefs highlighted further in outreach to caregivers and staff |
| 3. Successful outreach to some groups |  |
| 3a. Use of stories and testimonials | Caregiver materials expanded to include more diverse and sophisticated approaches   - Videos containing caregiver stories and testimonials - Emails and online landing page with caregiver quotes, pictures, and links to videos   New strategy of targeting staff   - Videos and emails with staff stories/testimonials on how workshop helps caregivers, reduces staff workload - Quarterly updates with new stories/materials “to keep it fresh” |
| 3b. Multiple contact episodes and materials | New use of national-level VA listservs and forums to increase number and type of contacts with caregivers   - Regular emails generated at national level to multiple VA listservs for caregivers - Workshop content demonstrated during national caregiver education conference calls |
| 4. Missed opportunities for improved outreach—suggesting need for the following |  |
| 4a. Detailed information on workshop content and structure | Details on workshop added to outreach materials for both caregivers and staff  New demo workshop created for staff where can explore/experience workshop structure and content |
| 4b. Expanded online mechanisms for outreach and enrollment | New Facebook caregiver group established where workshop alumni and other caregivers share information   - Social media stories/testimonials on workshop and instructions on how to register with VA   For caregivers already registered with VA, new emails with embedded web links allow them to self-enroll |
| 4c. Partnership with local communities and community groups | New marketing targeting external community groups   - Press releases that are community-facing - Outreach to Veteran service organizations |
| 4d. Increased outreach to certain caregiver groups and their health care teams | New internal marketing to local, regional, and national VA stakeholders   - Monthly emails to social workers and home visit nurses at local VA centers - Press releases with content designed for inclusion within internal communications/newsletters - CSP^b^ leadership talks on workshop given to leadership peers in social work and mental health |
| 5. Missed opportunities to support staff—suggesting need for the following |  |
| 5a. Training and mentoring for new staff | Recurrent trainings for new local staff every 6 months  Regular “reminder” emails to new staff about online library of outreach materials and how to refer caregivers |
| 5b. Improved data management capabilities to   - Generate outreach contacts - Track caregivers and outreach - Target follow-up outreach | Listserv outreach to caregivers as described above  Workshop modified to send local CSCs regular reports on caregivers (identity, whether self-referred/referred by others) and place in workshop process (registration, enrollment, completion)  Workshop modified to send repeated email invitations to caregivers who have registered but not enrolled |
| ^a^VA: US Department of Veterans Affairs.  ^b^CSP: Caregiver Support Program. | |
